# A randomised controlled single-centre open-label pharmacokinetic study to examine various approaches of nicotine delivery using electronic cigarettes

**DOI:** 10.1038/s41598-020-76610-4

**Published:** 2020-11-24

**Authors:** James K. Ebajemito, Michael McEwan, Nathan Gale, Oscar M. Camacho, George Hardie, Christopher J. Proctor

**Affiliations:** 1 British American Tobacco (Investments) Limited, Research and Development, Regents Park Road, Southampton, SO15 8TL UK; 2 DoctorProctorScience Ltd, 157 Cavendish Meads, Sunninghill, Ascot, SL5 9TG UK

**Keywords:** Pharmacology, Pharmacodynamics, Pharmacokinetics

## Abstract

Smokers who switch completely to e-cigarettes may reduce their relative risk of tobacco-related disease. Effective nicotine delivery from e-cigarettes is important in consumer acceptance. We assessed whether protonated nicotine and e-cigarette devices delivering greater aerosol mass increase nicotine delivery and product liking. A randomised controlled non-blinded eight-arm crossover study was used to assess plasma nicotine pharmacokinetics and product liking for two e-cigarettes (Vype ePen3 and Vype ePen) with various nicotine e-liquid formulations and a conventional cigarette among 24 healthy dual-users of cigarettes and e-cigarettes. Product use and puff count were also assessed. Results show that nicotine bioavailability was greater for Vype ePen3 with greater aerosol mass delivery than for Vype ePen (C_max_, *p* = 0.0073; AUC_0–120 min_, *p* = 0.0102). Protonated nicotine (18 mg/mL, medium protonation) e-liquid yielded higher nicotine bioavailability than unprotonated nicotine (18 mg/mL) e-liquid (C_max_, *p* = 0.0001; AUC_0–120 min_, *p* = 0.0026). There was no significant difference in T_max_ between e-liquids. Nicotine bioavailability did not differ between nicotine benzoate formulation (30 mg/mL nicotine, high protonation) and combustible cigarettes (C_max_, *p* = 0.79; AUC_0–120 min_, *p* = 0.13). Vype ePen3 with protonated nicotine delivers nicotine more efficiently with the potential to increase product liking relative to earlier devices using unprotonated e-liquid.

## Introduction

Accumulating scientific evidence shows that electronic nicotine delivery systems, such as e-cigarettes, may offer an effective alternative to continued smoking^1–4^. Despite the popularity of e-cigarettes as an alternative to cigarettes, a substantial proportion of adult smokers who try e-cigarettes often discontinue or use both e-cigarette and cigarettes concurrently (i.e., dual-use) due to reported lack of product acceptability^5–8^. A more acceptable e-cigarette product and/or e-liquid might contribute to an increase in sustained smoking abstinence in smoking populations or smokers with a desire and sufficient motivation to quit smoking.

Pharmacokinetic (PK) studies of conventional cigarette smoking have reported maximum plasma nicotine concentrations (C_max_) in the region of 10–21 ng/mL, depending on the product used^9–11^. Earlier generations of e-cigarette products have nicotine yields lower than that of a combustible cigarette^12–15^, which may be associated with a lower product acceptance. Therefore, an initial step to potentially improve the user acceptability of an e-cigarette product might be to match the nicotine delivery of a combustible cigarette during use.

An approach to increase nicotine delivery of e-cigarettes is to boost nicotine mass in the aerosol by increasing the power of the device. This has been shown to generate a larger volume of aerosol^16^, and in turn should also increase the bioavailability of nicotine for the user. However, nicotine is a well-known irritant of the pharynx^17^, and increasing nicotine levels in the inhaled aerosol can lead to acceptability issues such as harshness, irritancy, and coughing. Therefore, a range of approaches, in combination with higher nicotine levels, are needed to achieve both pharmacokinetic (PK) and product liking equivalent to a combustible cigarette. Recently, the use of nicotine salts have become common in commercial e-liquids. Unprotonated nicotine can be converted to nicotine salts by protonation via, for example, the addition of weak acids e.g. lactic and benzoic acid^11,17,18^. Aerosolised nicotine salts are less volatile than unprotonated nicotine^19,20^ and might be expected to be less irritating to the pharynx than equivalent concentrations of the more volatile unprotonated nicotine. Lower volatility may also facilitate greater lung exposure and absorption of nicotine rather than buccal absorption^21^, offering quicker and more efficient nicotine transport to the brain. Indeed, in the past year, two studies have demonstrated that e-cigarettes with nicotine salt formulations (nicotine lactate and nicotine benzoate) can achieve nicotine absorption equivalent to that of conventional cigarettes, although the nicotine e-liquid concentrations investigated were up to 48 mg/mL and 59 mg/mL, respectively^11,22^, which are considerably higher than the limits set by some regulatory bodies.

The aim of the present study was therefore to investigate different approaches to increasing nicotine delivery by e-cigarettes and their impact on product liking. Using a PK study to quantify plasma nicotine concentration following the use of e-cigarettes and combustible cigarettes, we compared different products and e-liquids to assess the effect of delivery of greater aerosol mass, protonated versus unprotonated nicotine, and increasing e-liquid nicotine concentration on both nicotine delivery and subjective consumer product liking. Given that smokers and e-cigarette users often use the products differently in terms, for example, of puff frequency and puff volume^23^, we used an ad libitum puffing regime to mimic real-life use as much as possible. However, because some studies have previously used a fixed puffing regime, the effect of puffing regime was also assessed to inform best practice for future studies. A summary of the key comparisons:

1. Comparison of plasma nicotine levels and product liking following use of protonated e-liquid with unprotonated e-liquid
2. Comparison of plasma nicotine delivery and product liking following use of a device with higher aerosol mass (Vype ePen 3) to its predecessor (Vype ePen).
3. Compare different levels of protonation (high, medium, low) and nicotine strengths (12, 18, 30 mg/mL) on plasma nicotine delivery and product liking.
4. Compare the pharmacokinetic profile of an ad libitum puffing regime to a fixed puffing regime.

## Methods

### Study design

This study was a randomised, single centre, non-blinded, eight-arm cross-over PK study registered in International Standard Randomised Controlled Trial Number (ISRCTN) database (ISRCTN: 55307091) and approved by the Wales Research Ethics Committee (REC reference: 18/WA/0353; 25th October 2018). The study was conducted in accordance with the International Council for Harmonisation (ICH) Guideline for Good Clinical Practice (GCP) and the Declaration of Helsinki. All subjects signed an informed consent form prior to any study procedures being performed. Subjects’ personal data were handled with strictest confidence in accordance with General Data Protection Regulation (GDPR; 2016/679).

Full information on the study design can be found in the protocol (Supplementary Information). In brief, participants attended the study centre on Day 1 for a 9-day confinement with eight PK sessions. The afternoon before each PK analysis, subjects underwent a familiarisation session, during which they were allowed to use their usual product, followed by the next day’s investigational product, giving it an initial rating for product liking. After a 12-h period of abstinence from nicotine or tobacco use, the PK session took place the following morning, during which the subjects used only the assigned investigational product as instructed, and PK and product liking analysis were carried out. On the morning of Day 1 after 12-h abstinence from nicotine, subjects were asked to smoke a single cigarette or use an e-cigarette, taking ad libitum or fixed puffs (10 puffs in total, one every 30 s) for a period of 5 min. Follow-up took place in the week after discharge (Supplementary Fig. 1). A summary of the study arms and corresponding investigational product parameters is presented in Table 1.

**Table 1:** Study arms and product characteristics.

Products	Short code	Article	Category	Brand	Nicotine conc. (mg/mL)	Protonation levels	Flavour	Puff protocol
A	N/A	Control	Cigarette	B&H Skyblue^a^	N/A	N/A	N/A	Ad libitum
B	N/A	Control	Cigarette	B&H Skyblue^a^	N/A	N/A	N/A	Fixed
C	ePen2.0BT18	Comparator	e-cigarette	Vype ePen	18	Unprotonated	Blended Tobacco	Ad libitum
D	ePen3.0BT18	Test #1	e-cigarette	Vype ePen3	18	Unprotonated	Blended Tobacco	Ad libitum
E	ePen3.0MB18VP	Test #2	e-cigarette	Vype ePen3	18	Medium protonation	MasterBlend Tobacco	Ad libitum
F	ePen3.0MB30VP	Test #3	e-cigarette	Vype ePen3	30	High protonation	MasterBlend Tobacco	Ad libitum
G	ePen3.0MB18VP	Test #4	e-cigarette	Vype ePen3	18	Medium protonation	MasterBlend Tobacco	Fixed
H	ePen3.0MB12VP	Test #1	e-cigarette	Vype ePen3	12	Low protonation	MasterBlend Tobacco	Ad libitum

*N/A* not applicable.

^a^Benson & Hedges Skyblue—7 mg ISO tar cigarette.

### Participants

The study recruited 24 healthy male and female volunteer subjects, who were aged between 19 and 60 years inclusive, and judged to be heathy at screening. All volunteers were considered “dual-users”; in other words, they were daily e-cigarette users and active smokers of combustible cigarettes and/or roll-your-own cigarettes (> 6 mg International Standard Organization [ISO] tar yield). In addition, they had smoked regularly for at least 1 year (maximum of 21 cigarettes per week) and were not currently attempting or planning to quit. Smoking and/or vaping status was confirmed by using a urinary cotinine level of ≥ 200 ng/ml (One Step cotinine test kit) at screening. All volunteers were informed that they were free to quit smoking/vaping and withdraw from the study at any time.

### Investigational products

Two e-cigarette devices were under investigation in this study. Vype ePen, a commercially available (at the time of study) closed-system e-cigarette with a silica rope wick, manufactured by British American Tobacco. Vype ePen consists of a reusable section (containing a 650 mAh rechargeable battery and an actuation button), a mouthpiece cover, and disposable cartridges. The device comes with two power settings (high, 4.4 W; and low, 2.8 W) and has been described in detail by Margham et al*.*^24^. In this study, subjects used Vype ePen at the high-power setting.

Vype ePen3 is a commercially sourced closed-system e-cigarette with a cotton wick, manufactured by British American Tobacco. It comprises a reusable section with a rechargeable 650 mAh battery and actuation button, and a disposable cartridge with integral mouthpiece. The device has a single 6 W power setting and delivers approximately twice the amount of vapour as the Vype ePen.

A Benson & Hedges Skyblue (Japan Tobacco International) 7 mg ISO tar yield combustible cigarette was used as a control product.

### Study outcome measures

#### Primary endpoint

##### Nicotine PK assessment

On each study day (Days 1–8), before, during and after product use, blood samples were collected either by direct venepuncture or from a cannula placed in a forearm vein, at the following times: 5 min before product use (– 5 min), and then at 1, 3, 5, 7, 9, 15, 30, 45, 60, 90 and 120 min. Heart rate was also measured at these times. Blood samples were collected into a lithium heparin monovette tube. No later than 60 min after collection, samples were centrifuged at 3500 RPM at 4 °C for 10 min. The plasma was transferred to two polypropylene screw cap tubes and stored at – 20 °C within 120 min of collection. Plasma nicotine analysis was performed by liquid chromatography with tandem mass spectrometry detection (LC-MS-MS) using the instrument in turbo ion spray, positive ion Multiple Reaction Monitoring (MRM) mode. The LC-MS-MS system consisted of an Applied Biosystems MDS Sciex API 4000 triple quadrupole atmospheric pressure ionization mass spectrometer. The lower limit of quantification (LLOQ) was determined to be 0.494 ng/ml (Seirian Laboratories, Merthyr Tydfil, UK).

#### Secondary endpoint

##### Product use assessment

Before and after use of each e-cigarette, the e-cigarettes (device and cartridge in situ) were weighed to determine the device mass loss (DML), which is the loss of mass (g) during the puffing period (pre- and post-cartridge weight). DML was used to calculate the amount of e-liquid used by each volunteer. Puff counts were also recorded during the use of all study products.

##### Subjective liking assessment

At − 5 min prior to product use, and 15 min and 120 min post product use, subjects completed a single item product liking questionnaire for the products that they used during each PK session. The assessment recorded prior to study product use was based on subjects’ experience during the familiarisation period, while the assessment during the PK session was based on the study product used during the PK session. The product satisfaction questionnaire consists of a 100 mm line visual analogue scale (VAS) on which subjects placed a vertical line indicating how much they like the nicotine product after being asked the question:

Can you tell me how much you like this nicotine product?

### Safety endpoints

The medical history, physical examination, vital signs and adverse events (AEs)/serious adverse events (SAEs) of study subjects were recorded during the study.

### Sample size calculation

From a previous pilot study, we found that the C_max_ least square mean of smoking subjects can reach values around 13–14 ng/mL nicotine in plasma and coefficient of variation (CVs) between 40 and 60% among the different arms (data not shown). Based on these data, a sample size calculation was performed using PROC POWER SAS 9.4 (SAS Institute, Cary NC) to assess superiority between ePen3 using an 18 mg/mL unprotonated e-liquid and and the same product and liquid but containing medium protonation benzoic acid. This comparison assumes superiority to have a ratio between the C_max_ > 1 with a ratio between means of 1.4, *β* = 0.2 and α = 0.05. Based on these assumptions, 22 subjects completing the study would be the minimum required to successfully demonstrate superiority, providing an actual power of 0.814.

### Randomisation

A randomisation scheme for study products use was produced using a computer-generated pseudo-random permutation procedure using SAS version 9.3 (SAS Institute Inc.). A randomisation code for 24 subjects was produced based on a Williams Latin Square design for an 8 × 8 crossover with 8 sequences, 3 subjects per sequence^25^. Due to the investigational products being visibly different, it was impossible to blind the assigned products.

### Statistical analysis

Subjects were assigned to the PK population set (PK set) if they completed the use of the study product, had data for the critical time points in the plasma nicotine concentration–time profiles, did not use a concomitant medication that rendered the concentration profile unreliable, and did not violate the protocol (major protocol deviation) in a way that might invalidate or bias the PK results.

The following PK parameters were calculated: C_max_, T_max_, AUC_0–120 min_. The derived PK endpoints were listed and summarised by product using the descriptive statistics N, n, arithmetic mean, geometric mean, arithmetic standard deviation (SD), CV%, minimum, median and maximum. Following logarithmic transformation, C_max_ and AUC_0–120 min_ values were subjected to an analysis of covariance (ANCOVA) including fixed effects for sequence, period and product, and a random effect of subject nested within sequence, with baseline %C_max_ concentration as a covariate. Point estimates and 95% confidence intervals (CI) were constructed for the contrasts of interest between each of the products. The point and interval estimates were back-transformed to provide estimates of the mean geometric ratios and corresponding 95% CI. In addition, estimated geometric means and 95% CI were presented for each product.

For the product liking assessment, the VAS scores were transformed (100—score) to range from 0 (Not at all) to 100 (Very much). Pre-study product use assessment was based on the product familiarisation period on the previous day. Post-study use was based on study product use during the PK session. The transformed product liking scores were listed by subject and summarised by product using descriptive statistics at each time point.

E-cigarette product usage data, including mass difference from e-cigarette DML and puff count were summarised for each product using descriptive statistics. The DML was calculated by subtracting the final device weight from the starting device weight.

Statistical analyses were performed using SAS Version 9.3 (SAS Institute, Cary NC).

## Results

### Study population demographics

Table 2 shows the demographic characteristics of the study participants. At screening and enrolment, all subjects were considered healthy. Of the 24 subjects enrolled in the study, 23 subjects completed the 8-day continuous enrolment with use of all test products and the follow-up (per-protocol population). One subject was withdrawn following eligibility check for smoking (subject smoked 23 cigarettes per week, not a maximum of 21) and vaping history (was not a daily user).

**Table 2:** Demographic characteristics of participants.

Variables	Characteristics^a^
Number of subjects (safety population)	24
Age (years)	37.3 ± 12.6
Sex (M:F)	15:9
Weight (Kg)	71.5 ± 13.48
Height (m)	1.70 ± 0.10
BMI (kg/m^2^)	24.8 ± 3.45

*M* Male; *F* Female; *BMI* body mass index.

^a^Data are mean ± SD.

### Nicotine pharmacokinetics

**Figure 1:**
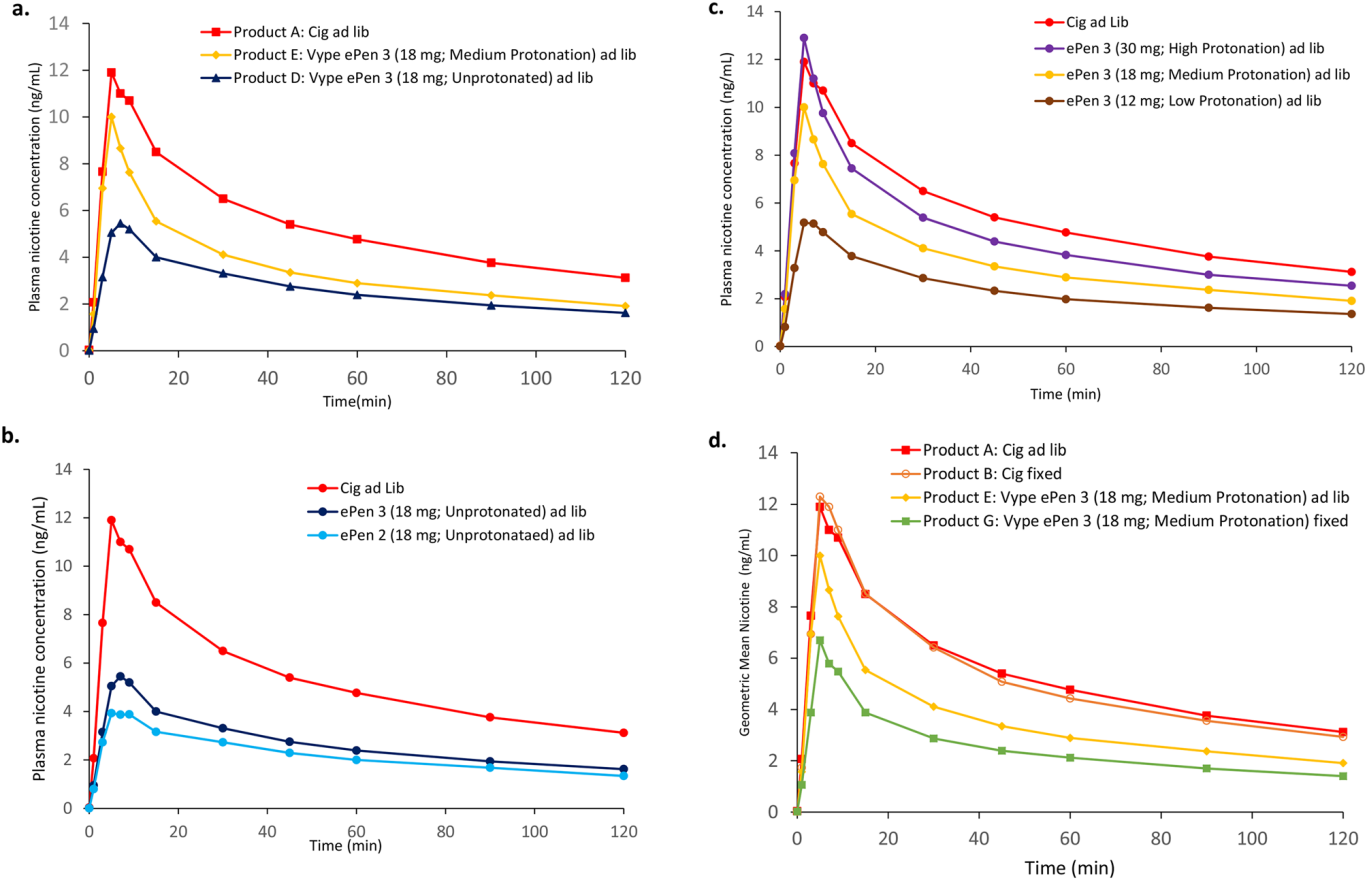
Pharmacokinetic profiles: mean plasma nicotine concentration of study products on a linear scale over 120 min (**a**) effect of nicotine salt (**b**) effect of device (**c**) effect of nicotine and protonation (acid) levels (**d**) effect of puffing protocol.

Nicotine PK data for all study arms is summarised below in Table 3. Regarding the effect of nicotine protonation in the e-liquid, nicotine benzoate e-liquid yielded a significantly higher nicotine bioavailability than unprotonated nicotine e-liquid (Product E (ePen3.0MB18VP) versus Product D (ePen3.0BT18), respectively; C_max_: *p* = 0.0001; AUC_0–120 min_: *p* = 0.0026). The geometric mean C_max_ was 10.8 ng/mL and 6.36 ng/mL, and mean AUC_0–120 min_ was 429 min * ng/mL and 325 min * ng/mL respectively (Table 3 and Fig. 1a). However, the nicotine protonation level of the e-liquid did not have a significant effect on T_max_ (Table 3).

**Table 3:** Arithmetic mean (95% CI) plasma nicotine concentration time profiles of investigational products (PK set).

Product^a^	Summary statistic	C_max_ (ng/ml)	T_max_ (min)	AUC_0–120 min_
A (N = 23)	Mean	18.5	7.22	717
	SD	12.5	5.67	241
	Geo. mean	14.5	N/A	660
	CV%	67.2	N/A	33.6
	Min	2.51	3.00	142
	Med	14.3	5.00	751
	Max	45.9	30.0	1010
B (N = 23)	Mean	16.3	7.00	666
	SD	10.4	2.34	212
	Geo. mean	13.7	N/A	631
	CV%	63.6	N/A	31.9
	Min	3.79	5.00	248
	Med	13.0	7.00	658
	Max	48.3	15.0	1160
C (N = 22)	Mean	5.82	12.2	298
	SD	3.81	10.3	137
	Geo. mean	4.79	N/A	267
	CV%	65.4	N/A	45.9
	Min	0.89	5.00	70.3
	Med	5.57	7.00	290
	Max	18.3	30.0	634
D (N = 23)	Mean	8.01	10.7	365
	SD	5.38	9.34	169
	Geo. mean	6.36	N/A	325
	CV%	67.2	N/A	46.3
	Min	1.32	3.00	87.8
	Med	7.38	7.00	349
	Max	24.5	30.0	756
E (N = 23)	Mean	12.5	6.04	478
	SD	6.81	2.33	222
	Geo. Mean	10.8	N/A	429
	CV%	54.7	N/A	46.5
	Min	2.84	3.00	173
	Med	9.93	5.00	436
	Max	32.5	15.0	924
F (N = 23)	Mean	16.8	5.70	628
	SD	9.24	2.53	294
	Geo. Mean	14.1	N/A	553
	CV%	55.1	N/A	46.9
	Min	4.08	3.00	139
	Med	16.3	5.00	588
	Max	39.2	15.0	1100
G (N = 22)	Mean	9.79	6.64	350
	SD	8.4	2.59	199
	Geo. Mean	7.16	N/A	303
	CV%	85.8	N/A	57.0
	Min	2.48	5.00	95.5
	Med	5.64	5.00	326
	Max	29.7	15.0	902
H (N = 23)	Mean	7.33	7.83	317
	SD	4.56	5.40	149
	Geo. Mean	5.97	N/A	284
	CV%	62.2	N/A	47.1
	Min	1.45	5.00	109
	Med	6.03	7.00	310
	Max	20.0	30.0	669

*N* number of subjects; *SD* standard deviation; *CV* coefficient of variation; *Geo. Mean* geometric mean.

^a^Products: A, combustible cigarette (ad libitum); B, combustible cigarette (fixed); C, ePen2.0BT18 (ad libitum); D, ePen3.0BT18 (ad libitum); E, ePen3.0MB18VP (ad libitum); F, ePen3.0MB30VP (ad libitum); G, -ePen3.0MB18VP (fixed); H, ePen3.0MB12VP (ad libitum). PK population dataset: of 24 subjects enrolled, one dropped out and one did not fulfil the study criteria for Products C and G (see “Methods”).

In terms of the effect of aerosol mass, nicotine bioavailability was significantly greater following use of Vype ePen3, which delivers greater aerosol mass, as compared with Vype ePen (Product D (ePen3.0BT18) versus Product C (ePen2.0BT18), respectively, C_max;_ *p* = 0.0073 and AUC_0–120 min;_ *p* = 0.0102). The mean C_max_ was 6.36 ng/mL and 4.79 ng/mL, and mean AUC_0–120 min_ was 325 min * ng/mL and 267 min * ng/mL respectively (Table 3 and Fig. 1b). By contrast, aerosol mass delivery by the e-cigarette did not have a significant effect on T_max_.

Increasing the levels of both nicotine and benzoic acid in the e-liquid resulted in an increase in plasma nicotine bioavailability as summarised in Table 3 and Fig. 1c, such that the C_max_ was significantly greater (*p* = 0.0331) with use of 30 mg/mL with high protonation (Product F: ePen3.0MB30VP) as compared with 18 mg/mL with medium protonation (Product E: ePen3.0MB18VP), which was in turn significantly greater (*p* < 0.0001) than 12 mg/ml nicotine with low protonation (Product H: ePen3.0MB12VP). Similarly, AUC_0–120 min_ was significantly greater (*p* = 0.0022) with use of 30 mg/mL nicotine with high protonation (Product F: ePen3.0MB30VP) compared to 18 mg/ml nicotine with medium protonation (Product E: ePen3.0MB18VP), which was in turn significantly greater (*p* < 0.0001) than 12 mg/mL nicotine with low protonation (Product H: ePen3.0MB12VP). Similar to the above findings, the differences in protonation and nicotine among the three products had no significant effect on T_max_ (Table 3).

Notably, the highest concentration of nicotine benzoate yielded a nicotine bioavailability that was not statistically different to that of the combustible cigarette control (Product F (ePen3.0MB30VP) vs. Product A (Cigarette ad libitum): C_max_; *p* = 0.79 and AUC_0–120 min_; *p* = 0.13; Table 3 and Fig. 1c). In addition, there was no significant difference in T_max_ between use of the Vype ePen3 with an e-liquid with high levels of protonated nicotine and smoking a combustible cigarette.

Lastly, we examined the effect of ad libitum versus fixed puffing regime on nicotine pharmacokinetics. Nicotine bioavailability following ad libitum use of Vype ePen3 was significantly higher than use under a fixed puffing regime of 1 puff every 30 s for 5 min (C_max;_ *p* = 0.0030 and AUC_0–120 min_; *p* = 0.0004). For the combustible cigarette, by contrast, different puffing protocols did not result in different nicotine PK profiles (Product A (Cigarette ad libitum) versus Product B (Cigarette fixed puff); Table 3 and Fig. 1d).

### Product use

Device mass loss (DML = pre–post product weight (g)) of the e-cigarette products and puff count of all study products during the PK session are reported in Table 5. Mean DML, indicating greater aerosol mass delivery, was highest for Product H, followed by Product E (ePen3.0MB18VP), Product D (ePen3.0BT18), Product F (ePen3.0MB30VP), Product C (ePen2.0BT18) and Product G (ePen3.0MB18VP), respectively. Puff count was highest for Product D (ePen3.0BT18), followed by Product C (ePen2.0BT18), Products E (ePen3.0MB18VP) and H (ePen3.0MB12VP) together, Product F(ePen3.0MB30VP) and Product G (ePen3.0MB18VP). The conventional cigarette, ad libitum and fixed puff use (Products A & B), were consumed in 11.50 ± 3.62 and 9.88 ± 0.45 puffs respectively.

### Product liking

Table 4 summarises the subjective product liking scores of all the study products. Pre- and post-product satisfaction was highest following use of the cigarette (ad libitum puffing), followed in order of highest liking by cigarette (fixed puffing), Vype ePen3 with 18 mg/mL nicotine with medium protonation and ad libitum puffing (Product E: ePen3.0MB18VP), fixed puffing (Product G: ePen3.0MB18VP), and without benzoic acid (Product D: ePen3.0BT18). Products H (ePen3.0MB12VP), F (ePen3.0MB30VP) and C (ePen2.0BT18) had the lowest pre and post-product use liking scores respectively.

**Table 4:** Summary of subjective product liking.

Products	Time point (min)	Mean	SD
A (N = 23)	− 5^a^	69.1	24.47
	15	68.6	26.16
	120	68.9	25.23
B (N = 24)	− 5^a^	64.7	25.99
	15	65.1	26.45
	120	65.4	26.87
C (N = 24)	− 5^a^	35.4	29.24
	15	35.6	29.54
	120	35.6	30.65
D (N = 24)	− 5^a^	53.9	27.86
	15	54.1	29.08
	120	55.7	29.24
E (N = 24)	− 5^a^	58	25.94
	15	61.5	22.3
	120	62.4	24.27
F (N = 24)	− 5^a^	46.7	26.47
	15	46.6	26.89
	120	45.1	29.24
G (N = 24)	− 5^a^	56	23.9
	15	58.7	24.43
	120	59.1	24.17
H (N = 23)	− 5^a^	52.6	24.1
	15	55	24.01
	120	55.1	24.12

Products: A, combustible cigarette (ad libitum); B, combustible cigarette (fixed); C, ePen2.0BT18 (ad libitum); D, ePen3.0BT18 (ad libitum); E, ePen3.0MB18VP (ad libitum); F, ePen3.0MB30VP (ad libitum); G, ePen3.0MB18VP (fixed); H, ePen3.0MB12VP (ad libitum).

*SD* standard deviation.

^a^– 5 min was a nominal time point corresponding to product satisfaction evaluated at the familiarisation session.

### Safety

Out of the 24 subjects enrolled on to the study, 13 experienced treatment emergent adverse events, out of which five where mild and eight were moderate. The most frequent AEs were cough and oropharyngeal pain. All AEs were resolved without treatment. No severe AEs or serious adverse events (SAEs) occurred and there were no reported discontinuations from the study due to AEs (Table 5).

**Table 5:** Summary product usage.

Product	Mean (± SD) puff count	Mean (± SD) DML
A (N = 23)	11.5 ± 3.62	N/A
B (N = 24)	9.9 ± 0.45	N/A
C (N = 24)	15.6 ± 4.90	0.0528 ± 0.02804
D (N = 24)	15.8 ± 5.00	0.0766 ± 0.03226
E (N = 24)	15.2 ± 5.76	0.0791 ± 0.03275
F (N = 24)	13.8 ± 4.19	0.0612 ± 0.02829
G (N = 24)	10.0 ± 0.00	0.0521 ± 0.02315
H (N = 23)	15.2 ± 4.92	0.0918 ± 0.03707

*N* Number of Subjects; *DML* Device Mass Loss; *SD* Standard Deviation; Product A—combustible cigarette (ad libitum); Product B—combustible cigarette (fixed); Product C—ePen2.0BT18 (ad libitum); Product D—ePen3.0BT18 (ad libitum); Product E—ePen3.0MB18VP (ad libitum); Product F—ePen3.0MB30VP (ad libitum); Project G—ePen3.0MB18VP (fixed); Product H—ePen3.0MB12VP (ad libitum).

## Discussion

Effective nicotine delivery and product liking from e-cigarettes play an important role in consumer acceptance. The present study compared nicotine bioavailability and product liking among two variants of an e-cigarette with different e-liquid formulations and a conventional cigarette. The principal aim of this study was to investigate nicotine bioavailability after the use of e-cigarettes varying in aerosol mass delivery and in nicotine concentration and/or presence of nicotine benzoate in the e-liquid, together with the effects on product liking.

The results show that Vype ePen3 has significantly higher C_max_ and AUC_0–120 min_ as compared with its predecessor Vype ePen (Fig. 1b). This was expected given the higher aerosol mass delivery of the Vype ePen3 device. Boosting nicotine mass in the aerosol by increasing the power of the device generates a larger volume of aerosol^16^, and in turn should also increase the bioavailability of nicotine for the user. This is also confirmed by the product usage data from this study (Table 5), which shows the puff count when used ad libitum was very similar for both products, but a greater DML was observed for Vype ePen3 as compared with Vype ePen. Notably, the product liking score for Vype ePen3 was approximately 40% higher than that of Vype ePen. Given that the e-liquid content was similar and the nicotine concentration identical, this increase in liking is most probably due to the higher aerosol mass delivery of Vype ePen3. Increasing nicotine delivery levels in the inhaled aerosol can lead to acceptability issues such as harshness, irritancy, and coughing^17^ and this is where aerosolised nicotine salts play a role in reducing irritation, even at high nicotine concentrations. In this study, nicotine benzoate increased nicotine bioavailability (Fig. 1c), and with higher levels of nicotine, this reached a level that was comparable to a combustible cigarette (30 mg/mL nicotine with high protonation variant only). This is in concordance with a previous study where lactic acid was used for nicotine protonation^11^; in that study, however, the investigational products did not achieve PK parity with the volunteers’ own brand of cigarette, despite nicotine concentrations of up to 48 mg/mL. The C_max_ of the combustible cigarette control used in this study resulted in a nicotine bioavailability that was lower than that observed in the O’Connell et al*.* study^11^, which is probably due to regional differences in cigarette product specification between the US and UK markets. However, our data are comparable to a previous study that used a 7 mg ISO tar cigarette^21^.

Product liking increased as nicotine bioavailability increased. The 12 mg/mL nicotine with low protonation and 18 mg/mL nicotine with medium protonation variants were comparable and slightly higher respectively, to the 18 mg/mL unprotonated nicotine variant in terms of product liking. However, this trend did not continue for the highest concentration of nicotine and benzoic acid. In combination with the comparatively lower product liking scores for the 30 mg/mL with high protonation variant (as compared with other nicotine with benzoic acid variants and the cigarette), the data showed that volunteers used less e-liquid and took fewer puffs on this particular product when used ad libitum (Table 5). This finding is interesting as it shows that there is a limit to the hypothesis that nicotine bioavailability drives product liking. A possible explanation, and a potential limitation of this study, might be that the study population was not acclimatised to e-liquids in excess of 20 mg/mL nicotine, irrespective of protonation. The EU limit of 20 mg/mL nicotine in a commercial e-cigarette product^26^ ensured that it is unlikely that UK vapers would typically use products in excess of 20 mg/mL. Given the well-known irritation effects of nicotine in the pharynx^23^, it is possible that volunteers found the experience of using higher strength nicotine uncomfortable.

Lastly, an ad libitum puffing protocol resulted in higher C_max_ and AUC_0–120 min_ compared to a fixed puff protocol with e-cigarette use but not with cigarette use (Fig. 1d). The findings with the combustible cigarette were expected, because volunteers were able to consume the entire cigarette within the allotted time frame. This is not the case with the e-cigarette, and the product use data (Table 5) demonstrated that a fixed puffing regime of 1 puff every 30 s for 5 min underestimates the real-life puffing frequency of experienced vapers. Furthermore, given the variability in the puff count for the ad libitum protocols, it is clear that different individuals use the product in different ways. This change in puffing frequency was associated with a corresponding increase in DML relative to the fixed puffing regime, indicating a greater consumption of e-liquid. This effect was also evident during cigarette consumption, where the mean puff count and variability of the ad libitum regime was greater than the fixed regime (Table 5). Previous studies have investigated both puffing regimes concurrently using different study designs (e.g., a few minutes of fixed use, followed by up to an hour of ad libitum use)^11,27,28^. Mixed results have been reported, which have been attributed to subjects changing their puffing behaviour and/or topography during product use. Therefore, ad libitum use is recommended for future PK studies, and other studies which aim to estimate effects of e-cigarettes in the general population.

Extrapolation of the findings from this study to dual users (e-cigarettes and cigarettes) or solus smokers in the general population may be constrained by a few limitations. While we appreciate that acute delivery of nicotine in a controlled environment provides useful insights into nicotine PK profiles following acute use, long-term ambulatory product use may differ. Additionally, due to the differences in puffing behaviour of dual users compared to solus smokers^29,30^, it is not clear if similar outcomes will be obtained in a population of solus smokers that are naïve to e-cigarette use. This study also precludes drawing any conclusions from the potential effect of e-liquid flavours, which previous studies have shown to influence product liking and use^31,32^. Although the e-liquid flavours used in the study were all tobacco-based flavours, they were not matched across all comparisons. Furthermore, as the tobacco and nicotine products used in this study are visibly different, blinding was not possible, which might have influenced the data to a certain degree. Lastly, although e-cigarette standards exist in various national jurisdictions^33,34^, all e-cigarettes are not the same; therefore, in principle, the findings from this study may not be replicated in other products.

## Conclusion

The PK and subjective data demonstrate that the Vype ePen3 device with greater aerosol mass delivery, coupled with a protonated nicotine e-liquid, delivers nicotine more efficiently and has the potential to increase product liking as compared with the earlier generation Vype ePen using unprotonated nicotine e-liquid. Greater e-cigarette product liking may increase smoking abstinence. Longer term studies are needed to determine whether product liking is maintained and results in improved smoking abstinence rates.

### Study registration

The study was registered in the ISRCTN database (ISRCTN: 55307091).

## Supplementary information

Supplementary Figures.
