# Supplementary material for: A randomised controlled single-centre open-label pharmacokinetic study to examine various approaches of nicotine delivery using electronic cigarettes
Source: Sci Rep. 2020 Nov 24;10:19980. doi: 10.1038/s41598-020-76610-4 (PMC7686355; doi:10.1038/s41598-020-76610-4)
Supplement: Supplementary file 1 — Supplementary Figures. [file 41598_2020_76610_MOESM1_ESM.docx]

**A Randomised Controlled Single-centre Open-label Pharmacokinetic Study to Examine Various Approaches of Nicotine Delivery Using Electronic Cigarettes**

James K. Ebajemito, Michael McEwan, Nathan Gale, Oscar M. Camacho, George Hardie and Christopher J. Proctor

# **SUPPLEMENTARY INFORMATION: TABLES, FIGURES & PROTOCOL**

**Supplementary Table 1:** Summary of Treatment Emergent Adverse Events (Safety Set)

|  | | | | | | | | | |
| --- | --- | --- | --- | --- | --- | --- | --- | --- | --- |
|  | **B&H Skyblue (Ad lib) (N=23)** | **B&H Skyblue (Fixed) (N=24)** | **EPEN2.0 BT18 (Ad lib) (N=24)** | **EPEN3.0 BT18 (Ad lib) (N=24)** | **EPEN3.0 MB18VP (Ad lib) (N=24)** | **EPEN3.0 MB30VP (Ad lib) (N=24)** | **EPEN3.0 MB18VP (Fixed) (N=24)** | **EPEN3.0 MB12VP (Ad lib) (N=23)** | **Overall (N=24)** |
|  | | | | | | | | | |
| **Number of TEAEs** | 4 | 5 | 5 | 1 | 5 | 2 | 3 | 3 | 28 |
|  | | | | | | | | | |
| **Number(%) of subjects reporting at least one:** |  |  |  |  |  |  |  |  |  |
| **TEAE** | 3 (13.0) | 5 (20.8) | 4 (16.7) | 1 (4.2) | 3 (12.5) | 2 (8.3) | 3 (12.5) | 3 (13.0) | 13 (54.2) |
| **Serious TEAE** | 0 (0.0) | 0 (0.0) | 0 (0.0) | 0 (0.0) | 0 (0.0) | 0 (0.0) | 0 (0.0) | 0 (0.0) | 0 (0.0) |
| **TEAE Leading to Withdrawal of IP** | 0 (0.0) | 0 (0.0) | 0 (0.0) | 0 (0.0) | 0 (0.0) | 0 (0.0) | 0 (0.0) | 0 (0.0) | 0 (0.0) |
|  | | | | | | | | | |
| **Number (%) of subjects with TEAE by severity:** |  |  |  |  |  |  |  |  |  |
| **Mild** | 1 (4.3) | 4 (16.7) | 1 (4.2) | 1 (4.2) | 1 (4.2) | 0 (0.0) | 2 (8.3) | 1 (4.3) | 5 (20.8) |
| **Moderate** | 2 (8.7) | 1 (4.2) | 3 (12.5) | 0 (0.0) | 2 (8.3) | 2 (8.3) | 1 (4.2) | 2 (8.7) | 8 (33.3) |
| **Severe** | 0 (0.0) | 0 (0.0) | 0 (0.0) | 0 (0.0) | 0 (0.0) | 0 (0.0) | 0 (0.0) | 0 (0.0) | 0 (0.0) |
|  |  |  |  |  |  |  |  |  |  |
| **Number (%) of subjects with TEAE by relationship to IP:** |  |  |  |  |  |  |  |  |  |
| **Not related** | 1 (4.3) | 3 (12.5) | 1 (4.2) | 0 (0.0) | 0 (0.0) | 0 (0.0) | 1 (4.2) | 1 (4.3) | 3 (12.5) |
| **Unlikely related** | 1 (4.3) | 1 (4.2) | 3 (12.5) | 1 (4.2) | 3 (12.5) | 2 (8.3) | 1 (4.2) | 1 (4.3) | 8 (33.3) |
| **Possibly related** | 1 (4.3) | 0 (0.0) | 0 (0.0) | 0 (0.0) | 0 (0.0) | 0 (0.0) | 0 (0.0) | 1 (4.3) | 1 (4.2) |
| **Related** | 0 (0.0) | 1 (4.2) | 0 (0.0) | 0 (0.0) | 0 (0.0) | 0 (0.0) | 1 (4.2) | 0 (0.0) | 1 (4.2) |
|  | | | | | | | | | |

## **Supplementary Table 2: Summary of Plasma Nicotine Concentration**

| Product |  | Time Point |  | n | Mean | Geo. Mean | SD | Minimum | Median | Maximum | CV (%) |
| --- | --- | --- | --- | --- | --- | --- | --- | --- | --- | --- | --- |
| B&H Skyblue (Ad lib) (N=23) |  | Pre-Dose -5 min |  | 23 | 0.500 | 0.0333 | 0.609 | 0.00 | 0.504 | 2.46 | 121.8 |
|  |  | 1 min |  | 23 | 3.85 | 2.07 | 5.36 | 0.247 | 1.53 | 24.9 | 139.3 |
|  |  | 3 mins |  | 23 | 12.2 | 7.66 | 11.6 | 0.955 | 7.69 | 45.9 | 95.5 |
|  |  | 5 mins |  | 23 | 15.9 | 11.9 | 11.6 | 2.19 | 14.3 | 43.9 | 72.8 |
|  |  | 7 mins |  | 22 | 13.2 | 11.0 | 7.58 | 2.29 | 11.7 | 30.2 | 57.3 |
|  |  | 9 mins |  | 23 | 12.1 | 10.7 | 5.48 | 2.41 | 11.9 | 24.4 | 45.2 |
|  |  | 15 mins |  | 23 | 9.30 | 8.50 | 3.37 | 2.13 | 8.98 | 14.9 | 36.3 |
|  |  | 30 mins |  | 23 | 7.06 | 6.50 | 2.37 | 1.34 | 7.18 | 10.4 | 33.6 |
|  |  | 45 mins |  | 23 | 5.86 | 5.40 | 1.96 | 1.03 | 6.11 | 8.69 | 33.4 |
|  |  | 60 mins |  | 23 | 5.23 | 4.77 | 1.91 | 1.00 | 5.72 | 8.40 | 36.6 |
|  |  | 90 mins |  | 23 | 4.09 | 3.76 | 1.52 | 0.911 | 4.25 | 6.84 | 37.2 |
|  |  | 120 mins |  | 23 | 3.48 | 3.12 | 1.47 | 0.726 | 3.43 | 6.22 | 42.2 |
| B&H Skyblue (Fixed) (N=23) |  | Pre-Dose -5 min |  | 23 | 0.406 | 0.0179 | 0.556 | 0.00 | 0.00 | 2.09 | 136.9 |
|  |  | 1 min |  | 23 | 2.33 | 1.73 | 1.80 | 0.247 | 1.63 | 6.75 | 77.4 |
|  |  | 3 mins |  | 23 | 8.66 | 6.94 | 5.92 | 1.69 | 6.92 | 23.3 | 68.3 |
|  |  | 5 mins |  | 23 | 15.0 | 12.3 | 10.4 | 3.47 | 11.9 | 48.3 | 69.6 |
|  |  | 7 mins |  | 23 | 13.6 | 11.9 | 7.23 | 3.28 | 13.0 | 33.0 | 53.3 |
|  |  | 9 mins |  | 23 | 12.2 | 11.0 | 5.66 | 3.54 | 11.6 | 28.1 | 46.5 |
|  |  | 15 mins |  | 23 | 9.10 | 8.52 | 3.32 | 3.57 | 8.92 | 17.3 | 36.5 |
|  |  | 30 mins |  | 23 | 6.74 | 6.42 | 2.00 | 2.45 | 6.65 | 10.4 | 29.7 |
|  |  | 45 mins |  | 23 | 5.34 | 5.08 | 1.64 | 2.07 | 5.31 | 8.33 | 30.7 |
|  |  | 60 mins |  | 23 | 4.67 | 4.43 | 1.52 | 2.12 | 4.57 | 7.64 | 32.6 |
|  |  | 90 mins |  | 23 | 3.79 | 3.56 | 1.28 | 1.47 | 3.76 | 5.94 | 33.8 |
|  |  | 120 mins |  | 23 | 3.13 | 2.93 | 1.08 | 1.16 | 3.01 | 5.23 | 34.6 |

| Product |  | Time Point |  | n | Mean | Geo. Mean | SD | Minimum | Median | Maximum | CV (%) |
| --- | --- | --- | --- | --- | --- | --- | --- | --- | --- | --- | --- |
| EPEN2.0BT18 (Ad lib) (N=22) |  | Pre-Dose -5 min |  | 22 | 0.213 | 0.00505 | 0.389 | 0.00 | 0.00 | 1.32 | 182.4 |
|  |  | 1 min |  | 22 | 1.18 | 0.799 | 0.984 | 0.247 | 0.945 | 3.41 | 83.6 |
|  |  | 3 mins |  | 22 | 3.80 | 2.73 | 3.17 | 0.577 | 3.19 | 12.5 | 83.5 |
|  |  | 5 mins |  | 22 | 5.24 | 3.93 | 4.09 | 0.856 | 4.61 | 18.3 | 78.0 |
|  |  | 7 mins |  | 22 | 4.67 | 3.87 | 2.65 | 0.891 | 5.15 | 10.3 | 56.9 |
|  |  | 9 mins |  | 22 | 4.57 | 3.88 | 2.36 | 0.735 | 4.62 | 9.41 | 51.7 |
|  |  | 15 mins |  | 22 | 3.56 | 3.16 | 1.63 | 0.712 | 3.52 | 7.31 | 46.0 |
|  |  | 30 mins |  | 22 | 3.06 | 2.73 | 1.44 | 0.695 | 2.81 | 6.34 | 46.9 |
|  |  | 45 mins |  | 22 | 2.57 | 2.29 | 1.17 | 0.653 | 2.37 | 5.28 | 45.5 |
|  |  | 60 mins |  | 22 | 2.23 | 2.00 | 1.05 | 0.625 | 2.06 | 5.05 | 46.8 |
|  |  | 90 mins |  | 22 | 1.89 | 1.68 | 0.864 | 0.582 | 1.86 | 3.77 | 45.7 |
|  |  | 120 mins |  | 22 | 1.54 | 1.34 | 0.734 | 0.247 | 1.59 | 3.32 | 47.7 |

| EPEN3.0BT18 (Ad lib) (N=23)` |  | Pre-Dose -5 min |  | 23 | 0.359 | 0.0128 | 0.554 | 0.00 | 0.00 | 2.06 | 154.4 |
| --- | --- | --- | --- | --- | --- | --- | --- | --- | --- | --- | --- |
|  |  | 1 min |  | 23 | 1.74 | 0.948 | 2.17 | 0.247 | 0.980 | 7.97 | 124.9 |
|  |  | 3 mins |  | 23 | 4.80 | 3.15 | 3.82 | 0.247 | 4.69 | 13.2 | 79.5 |
|  |  | 5 mins |  | 23 | 6.95 | 5.05 | 5.57 | 1.02 | 5.87 | 24.5 | 80.1 |
|  |  | 7 mins |  | 23 | 6.81 | 5.45 | 4.35 | 1.24 | 7.17 | 19.6 | 63.9 |
|  |  | 9 mins |  | 23 | 6.35 | 5.20 | 3.73 | 1.10 | 5.95 | 16.8 | 58.8 |
|  |  | 15 mins |  | 23 | 4.62 | 4.00 | 2.36 | 0.976 | 4.75 | 11.5 | 51.0 |
|  |  | 30 mins |  | 23 | 3.67 | 3.31 | 1.63 | 0.924 | 3.46 | 8.15 | 44.5 |
|  |  | 45 mins |  | 23 | 3.04 | 2.75 | 1.32 | 0.746 | 2.93 | 6.32 | 43.3 |
|  |  | 60 mins |  | 23 | 2.66 | 2.39 | 1.18 | 0.647 | 2.57 | 5.47 | 44.2 |
|  |  | 90 mins |  | 23 | 2.19 | 1.94 | 1.07 | 0.569 | 1.97 | 4.35 | 49.0 |
|  |  | 120 mins |  | 23 | 1.81 | 1.62 | 0.884 | 0.540 | 1.64 | 3.70 | 48.8 |

| Product |  | Time Point |  | n | Mean | Geo. Mean | SD | Minimum | Median | Maximum | CV (%) |
| --- | --- | --- | --- | --- | --- | --- | --- | --- | --- | --- | --- |
| EPEN3.0MB18VP (Ad lib) (N=23) |  | Pre-Dose -5 min |  | 23 | 0.412 | 0.0235 | 0.515 | 0.00 | 0.00 | 1.95 | 124.8 |
|  |  | 1 min |  | 23 | 2.64 | 1.57 | 2.62 | 0.247 | 1.41 | 8.50 | 99.4 |
|  |  | 3 mins |  | 23 | 8.10 | 6.95 | 4.28 | 1.41 | 6.66 | 19.9 | 52.9 |
|  |  | 5 mins |  | 23 | 11.8 | 10.0 | 6.82 | 2.41 | 9.93 | 32.5 | 57.7 |
|  |  | 7 mins |  | 23 | 10.1 | 8.66 | 5.51 | 2.84 | 8.68 | 21.7 | 54.4 |
|  |  | 9 mins |  | 23 | 8.74 | 7.63 | 4.46 | 2.61 | 7.93 | 17.5 | 51.1 |
|  |  | 15 mins |  | 23 | 6.14 | 5.54 | 2.72 | 2.21 | 5.84 | 11.2 | 44.2 |
|  |  | 30 mins |  | 23 | 4.62 | 4.11 | 2.25 | 1.46 | 3.74 | 9.58 | 48.7 |
|  |  | 45 mins |  | 23 | 3.84 | 3.35 | 2.02 | 1.13 | 3.54 | 8.28 | 52.6 |
|  |  | 60 mins |  | 23 | 3.30 | 2.89 | 1.70 | 0.928 | 3.11 | 6.73 | 51.3 |
|  |  | 90 mins |  | 23 | 2.73 | 2.37 | 1.49 | 0.861 | 2.50 | 5.79 | 54.5 |
|  |  | 120 mins |  | 23 | 2.22 | 1.91 | 1.22 | 0.624 | 1.99 | 5.03 | 55.1 |

| EPEN3.0MB30VP (Ad lib) (N=23) |  | Pre-Dose -5 min |  | 23 | 0.492 | 0.0253 | 0.633 | 0.00 | 0.00 | 2.25 | 128.6 |  |
| --- | --- | --- | --- | --- | --- | --- | --- | --- | --- | --- | --- | --- |
|  |  | 1 min |  | 22 | 4.12 | 2.19 | 6.27 | 0.247 | 1.57 | 27.7 | 152.0 |  |
|  |  | 3 mins |  | 23 | 10.6 | 8.08 | 6.84 | 1.74 | 9.18 | 23.6 | 64.6 |  |
|  |  | 5 mins |  | 23 | 15.6 | 12.9 | 9.22 | 4.08 | 15.9 | 39.2 | 59.1 |  |
|  |  | 7 mins |  | 23 | 13.3 | 11.2 | 6.93 | 2.89 | 14.1 | 25.8 | 52.2 |  |
|  |  | 9 mins |  | 23 | 11.5 | 9.76 | 5.88 | 2.35 | 10.9 | 24.5 | 51.3 |  |
|  |  | 15 mins |  | 23 | 8.53 | 7.45 | 4.02 | 1.82 | 7.95 | 15.2 | 47.1 |  |
|  |  | 30 mins |  | 23 | 6.17 | 5.39 | 3.00 | 1.42 | 5.15 | 12.2 | 48.7 |  |
|  |  | 45 mins |  | 23 | 5.04 | 4.39 | 2.49 | 1.17 | 4.70 | 9.63 | 49.4 |  |
|  |  | 60 mins |  | 23 | 4.43 | 3.83 | 2.27 | 0.931 | 3.80 | 8.27 | 51.1 |  |
|  |  | 90 mins |  | 23 | 3.55 | 3.00 | 1.96 | 0.604 | 3.08 | 7.05 | 55.3 |  |
|  |  | 120 mins |  | 22 | 2.99 | 2.54 | 1.62 | 0.582 | 2.30 | 5.59 | 54.2 |  |
| \| Product \|  \| Time Point \|  \| n \| Mean \| Geo. Mean \| SD \| Minimum \| Median \| Maximum \| CV (%) \| \| --- \| --- \| --- \| --- \| --- \| --- \| --- \| --- \| --- \| --- \| --- \| --- \| \| EPEN3.0MB18VP (Fixed) (N=22) \|  \| Pre-Dose -5 min \|  \| 22 \| 0.406 \| 0.0204 \| 0.500 \| 0.00 \| 0.00 \| 1.54 \| 123.1 \| \|  \| 1 min \|  \| 22 \| 2.19 \| 1.06 \| 3.11 \| 0.247 \| 0.946 \| 13.0 \| 142.1 \| \|  \| 3 mins \|  \| 22 \| 5.75 \| 3.88 \| 5.67 \| 1.33 \| 3.08 \| 19.5 \| 98.7 \| \|  \| 5 mins \|  \| 22 \| 9.52 \| 6.70 \| 8.58 \| 2.13 \| 5.04 \| 29.7 \| 90.1 \| \|  \| 7 mins \|  \| 22 \| 7.63 \| 5.79 \| 6.25 \| 2.04 \| 4.39 \| 23.8 \| 81.9 \| \|  \| 9 mins \|  \| 22 \| 6.68 \| 5.48 \| 4.70 \| 2.33 \| 4.44 \| 18.9 \| 70.4 \| \|  \| 15 mins \|  \| 22 \| 4.43 \| 3.88 \| 2.28 \| 1.26 \| 3.68 \| 9.23 \| 51.5 \| \|  \| 30 mins \|  \| 21 \| 3.26 \| 2.87 \| 1.81 \| 0.988 \| 3.24 \| 8.96 \| 55.3 \| \|  \| 45 mins \|  \| 22 \| 2.74 \| 2.39 \| 1.53 \| 0.735 \| 2.52 \| 7.39 \| 55.6 \| \|  \| 60 mins \|  \| 22 \| 2.39 \| 2.12 \| 1.26 \| 0.696 \| 2.27 \| 6.32 \| 52.6 \| \|  \| 90 mins \|  \| 22 \| 1.95 \| 1.70 \| 1.10 \| 0.499 \| 1.74 \| 5.39 \| 56.5 \| \|  \| 120 mins \|  \| 22 \| 1.62 \| 1.40 \| 0.874 \| 0.247 \| 1.42 \| 4.17 \| 53.9 \| \| \| EPEN3.0MB12VP (Ad lib) (N=23) \|  \| Pre-Dose -5 min \|  \| 23 \| 0.347 \| 0.0128 \| 0.510 \| 0.00 \| 0.00 \| 1.93 \| 147.2 \| \| --- \| --- \| --- \| --- \| --- \| --- \| --- \| --- \| --- \| --- \| --- \| --- \| \|  \| 1 min \|  \| 23 \| 1.27 \| 0.818 \| 1.29 \| 0.247 \| 0.892 \| 5.77 \| 102.3 \| \|  \| 3 mins \|  \| 23 \| 4.68 \| 3.28 \| 3.82 \| 0.247 \| 3.79 \| 16.8 \| 81.5 \| \|  \| 5 mins \|  \| 23 \| 6.70 \| 5.18 \| 4.74 \| 1.18 \| 5.58 \| 20.0 \| 70.8 \| \|  \| 7 mins \|  \| 23 \| 6.18 \| 5.14 \| 3.52 \| 1.29 \| 5.60 \| 12.9 \| 57.0 \| \|  \| 9 mins \|  \| 22 \| 5.60 \| 4.78 \| 3.01 \| 1.45 \| 5.08 \| 11.6 \| 53.8 \| \|  \| 15 mins \|  \| 23 \| 4.29 \| 3.78 \| 2.08 \| 1.23 \| 4.08 \| 8.89 \| 48.4 \| \|  \| 30 mins \|  \| 23 \| 3.19 \| 2.86 \| 1.54 \| 1.13 \| 3.13 \| 7.01 \| 48.2 \| \|  \| 45 mins \|  \| 23 \| 2.57 \| 2.33 \| 1.21 \| 1.05 \| 2.49 \| 5.86 \| 46.9 \| \|  \| 60 mins \|  \| 23 \| 2.20 \| 1.98 \| 1.08 \| 0.875 \| 2.06 \| 5.58 \| 49.1 \| \|  \| 90 mins \|  \| 23 \| 1.81 \| 1.62 \| 0.904 \| 0.750 \| 1.64 \| 4.41 \| 50.0 \| \|  \| 120 mins \|  \| 23 \| 1.51 \| 1.36 \| 0.759 \| 0.639 \| 1.26 \| 3.83 \| 50.2 \| \| \| \| \| \| \| \| \| \| \| \| \| \| | | | | | | | | | | | | |

**
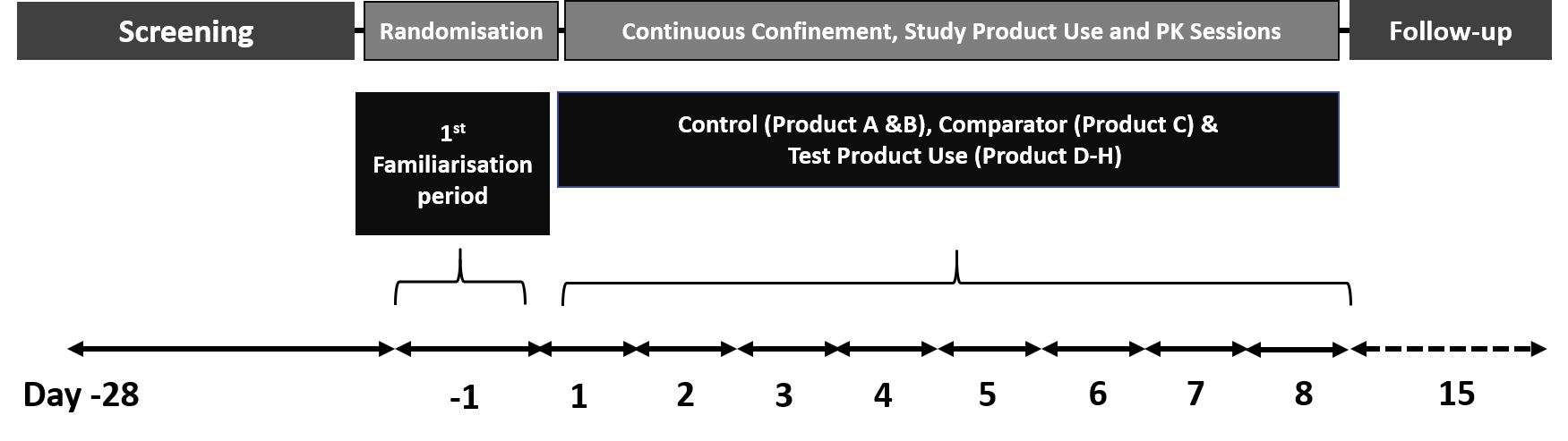
**

**Supplementary Figure 1:** Study design schematic. Screening took place within 28 days of enrolment (Day-1). Upon admission, subjects were randomised and provided with study products for familiarisation before each pharmacokinetic session. During the PK sessions on days 1-8, subjects used their randomly-assigned e-cigarette (Product C-H) or combustible cigarette (Product A or B) for pharmacokinetic and product satisfaction analysis. Follow up took place within a week after discharge.

**Protocol**

**A Randomised Controlled Single-centre Open-label Pharmacokinetic Study to Examine Nicotine Delivery from Different Variants of an E-cigarette and a Conventional Cigarette in Healthy subjects.**

Study number BAT4118015

Study Products Vype ePen 2 e-cigarette

Vype ePen 3 e-cigarette

B&H Skyblue Combustible cigarette

Sponsor British American Tobacco (Investments) Limited

**LIST OF ABBREVIATIONS AND DEFINITION OF TERMS**

| AE | Adverse event |
| --- | --- |
| ANCOVA | Analysis of covariance |
| AUC | Area under the curve |
| BAT | British American Tobacco |
| BMI | Body mass index |
| CI | Confidence interval |
| C_max_ | Maximum observed plasma concentration |
| CPD | Cigarettes per day |
| CRA | Clinical Research Associate |
| CRF | Case Report Form |
| CRO | Clinical Research Organisation |
| CRU | Clinical Research Unit |
| CSR | Clinical Study Report |
| CV% | Coefficient of variation |
| CYP | Cytochrome P450 |
| DML | Device mass loss |
| EC | Ethics Committee |
| ECG | Electrocardiogram |
| ENDS | Electronic nicotine delivery system |
| FSH | Follicle-stimulating hormone |
| FTND | Fagerstrom Test for Nicotine Dependence |
| GCP | Good Clinical Practice |
| HBsAg | Hepatitis B surface antigen |
| IB | Investigators Brochure |
| ICF | Informed consent form |
| ICH | International Council on Harmonisation |
| IP | Investigational products |
| IRB | Institutional Review Board |
| ISF | Investigator site file |
| ISRCTN | International Standard Registered Clinical/Social Study Number |
| kg | Kilogram |
| kg/m^2^ | Kilogram / metres squared |
| LC-MS/MS | Liquid chromatography-tandem mass spectrometry |
| MedDRA | Medical Dictionary for Regulatory Activities |
| MEq | Milliequivalents |
| MCH | Mean cell haemoglobin |
| MCHC | Mean cell haemoglobin concentration |
| MCV | Mean cell volume |
| mg/cig | Milliigrams / cigarette |
| mg/mL | Milligrams / millilitre |
| mL | Millilitre |
| MLE | Mouth level exposure |
| n | Number of subjects with non-missing observations |
| N | Number of subjects in the analysis population |
| Ng/mL | Nanogram / millilitre |
| NGP | Next generation product |
| NRT | Nicotine replacement therapy |
| OTC | Over-the-counter |
| PCV | Packed cell volume |
| PI | Principal Investigator |
| PK | Pharmacokinetic |
| RBC | Red blood cell |
| REC | Research Ethics Committee |
| RPM | Revolutions per minute |
| RYO | Roll your own |
| SAE | Serious adverse event |
| SAS | Statistical Analysis System |
| SD | Standard deviation |
| SOC | System organ class |
| SOP | Standard operating procedure |
| SUSAR | Suspected unexpected serious adverse reaction |
| TEAE | Treatment Emergent Adverse Event |
| T_max_ | Time to maximum concentration |
| TMF | Trial Master File |
| UK | United Kingdom |
| VID | Volunteer Information Document |
| W | Watt |
| WBC | White blood cell |
| WHO | World Health Organisation |

**STUDY SYNOPSIS**

| **Title of Study:** | A Randomised Controlled Single-centre Open-label Pharmacokinetic Study to Examine Nicotine Delivery from Different Variants of an E-cigarette and a Conventional Cigarette in Healthy subjects. |
| --- | --- |
| **Objectives:** | **Primary Objectives:**   - To determine kinetics of nicotine absorption into the blood of subjects using different variants of e-cigarettes and a cigarette. - To compare nicotine delivery from different e-cigarettes to one another, and with that from a conventional combustible cigarette. - Compare the effect of *ad libitum* puffing versus fixed puffing regime.   **Secondary Objectives:**   - Monitor heart rate during product use - Evaluate product satisfaction with different e-cigarettes and a conventional cigarette - Assess product use - Monitor safety profile of subjects using the study products   **Primary Endpoints:**   - Plasma nicotine C_max_, T_max_ and AUC_0-120_   **Secondary Endpoint:**   - Heart rate - Product satisfaction scores - Product use: device mass loss (DML), puff number and use count   **Other measures:**   - Mouth level exposure (MLE)   **Safety Endpoints:**   - Medical history of study subjects - Physical examination - Vital signs - Adverse events (AE)/serious adverse events (SAE) recording |
| **Study Design:** | This will be a randomised, non-blinded pharmacokinetic study of nicotine-containing products carried out in 24 healthy adult subjects.   - Subjects will attend the clinic site for a pre-study screening visit within 28 days of entry into the study. - Subjects who satisfy the inclusion/exclusion criteria will be entered into the study. - Eligible subjects will be admitted into the clinic for 9 days of continuous confinement, starting admission on the morning of Day -1. Upon admission, continued eligibility will be confirmed. In accordance with the study randomisation the subjects will be given the investigational product for familiarisation. - The number of conventional cigarettes smoked, e-liquid cartridges used and device mass loss (DML) will be recorded during the familiarisation period. - On the study days 1 to 8, after a minimum of 12 hours nicotine abstinence, subjects will smoke a regular (combustible) cigarette or use an e-cigarette *ad libitum* (with puffs counted) or fixed, and blood samples will be obtained for plasma nicotine analysis. - Blood samples will be taken at -5, 1, 3, 5, 7, 9, 15, 30, 45, 60, 90, 120 minutes relative to the first puff on a cigarette/e‑cigarette. Subjective questionnaires will also be provided for completion at pre-determined intervals during the PK session. - After each product use, used e-cigarette devices will be returned to the pharmacy, cut cigarette filters will be stored securely within the clinical unit. - Product Satisfaction Questionnaire will be completed during the product use sessions and heart (pulse) rate will also be measured throughout the PK assessment period. - No later than one week after discharge from clinic, a post-study follow-up will be performed, which will be conducted via a telephone call with the subjects. |
| **Number and type of Subjects:** | In total, 24 volunteers will be enrolled for 22 to complete, Adult male or female subjects who are current daily e-cigarette users (at least 18 mg/mL e-liquid) and active smokers (maximum of 21 cigarettes per week) of combustible cigarettes and/or roll your own cigarettes (>6 ISO tar). Subjects will be aged 19-60 inclusive and judged to be healthy by pre-study screening. |
| **Test Products:** | \| Products \| Product Code \| Product & Manufacturer \| Category \| E-liquid Nicotine Conc. (mg/mL) \| Protonation Levels \| Flavour \| Puff regime \| \| --- \| --- \| --- \| --- \| --- \| --- \| --- \| --- \| \| A \| N/A \| B&H Skyblue*; JTI \| Cigarette \| N/A \| N/A \| N/A \| *Ad lib* \| \| B \| N/A \| B&H Skyblue*; JTI \| Cigarette \| N/A \| N/A \| N/A \| Fixed \| \| C \| EPEN2.0BT18 \| Vype ePen 2; BAT \| e-cigarette \| 18 \| Unprotonated \| Blended Tobacco \| *Ad lib* \| \| D \| EPEN3.0BT18 \| Vype ePen 3; BAT \| e-cigarette \| 18 \| Unprotonated \| Blended Tobacco \| *Ad lib* \| \| E \| EPEN3.0MB18VP \| Vype ePen 3; BAT \| e-cigarette \| 18 \| Medium Protonation \| MasterBlend \| *Ad lib* \| \| F \| EPEN3.0MB30VP \| Vype ePen 3; BAT \| e-cigarette \| 30 \| High protonation \| MasterBlend \| *Ad lib* \| \| G \| EPEN3.0MB18VP \| Vype ePen 3; BAT \| e-cigarette \| 18 \| Medium Protonation \| MasterBlend \| Fixed \| \| H \| EPEN3.0MB12VP \| Vype ePen 3; BAT \| e-cigarette \| 12 \| Low Protonation \| MasterBlend \| *Ad lib* \|   *7mg/cig (ISO ‘tar); JTI – Japan Tobacco International; BAT – British American Tobacco; N/A – Not applicable |
| **Duration of Study:** | **Screening Period:** 28 days for all screening to take place.  **Study Duration:** Confined study (9 days per subject) with at least 12 hours between each PK session. |
| **Criteria for Evaluation:** | PK parameters will be calculated based on the plasma nicotine concentrations using samples collected during the study, following *ad libitum* or fixed single product use for a period of no longer than 5 minutes, after at least 12 hours of smoking cessation. Plots of blood nicotine concentration over time will be used to determine C_max_, T_max_ and AUC_0-120h_.  The safety evaluations for this study will include AEs, vital signs,and, physical examinations. |
| **Statistical Methods:** | Descriptive statistics (n, arithmetic mean, geometric mean standard deviation, CV(%), median, minimum and maximum) will be calculated for the PK parameters, as appropriate. Group means for each test product and each parameter will be compared using appropriate statistical tests. |

**STUDY PLAN**

**SCHEDULE OF ASSESSMENT**

|  | **Screening Visit** | **Admission**  **(Day -1)** | **Study Phase**  **(Day 1 to 8)** | **Discharge^^[[1]](#footnote-1)^^** | **Follow-up phone call** |
| --- | --- | --- | --- | --- | --- |
| Informed consent | X |  |  |  |  |
| Inclusion/exclusion criteria | X | X |  |  |  |
| Socio-demographic data | X |  |  |  |  |
| Tobacco, e-cigarette and nicotine use history questionnaire | X |  |  |  |  |
| FTND questionnaire | X |  |  |  |  |
| Urinary cotinine screen | X | X |  |  |  |
| Height, weight, BMI | X |  |  |  |  |
| Medical history | X | X |  |  |  |
| Adverse event recording | X | X | X | X | X |
| Vital signs | X | X |  | X |  |
| Physical examination | X | X^^[[2]](#footnote-2)^^ |  | X^2^ |  |
| ECG | X |  |  |  |  |
| Lung function test (without bronchodilator)^^[[3]](#footnote-3)^^ | X^3^ | X^3^ |  |  |  |
| Biochemistry, haematology, urinalysis and serology | X |  |  |  |  |
| Urine drugs of abuse and alcohol screen^^[[4]](#footnote-4)^^ | X | X |  |  |  |
| Pregnancy test^^[[5]](#footnote-5)^^ | X | X |  | X |  |
| FSH | X |  |  |  |  |
| Concomitant medication | X | X | X | X | X |
| Pharmacokinetics: Blood sampling (12 samples/assessment) |  |  | X |  |  |
| Product Usage (Use count, puff count, cartridge weight and MLE)^5^ |  | X | X |  |  |
| Heart (pulse) rate |  | X | X |  |  |
| Product Satisfaction Questionnaire^6^ |  |  | X |  |  |

**TIMINGS OF QUESTIONNAIRE ASSESSMENT, HEART RATE AND PK BLOOD DRAWS**

| **Time point (min)** | **Product Satisfaction** | **Heart Rate** | **PK Blood Draws** |
| --- | --- | --- | --- |
| -5 (prior to IP Use) | X | X | X |
| 1 |  | X | X |
| 3 |  | X | X |
| 5 |  | X | X |
| 7 |  | X | X |
| 9 |  | X | X |
| 15 | X | X | X |
| 30 |  | X | X |
| 45 |  | X | X |
| 60 |  | X | X |
| 90 |  | X | X |
| 120 | X | X | X |

**1. INTRODUCTION**

**1.1 BACKGROUND INFORMATION**

**1.1.1 Smoking and health**

Smoking has been identified as a contributing factor to numerous human disorders including lung cancer, chronic obstructive pulmonary disease, and atherosclerotic cardiovascular disease. The health risks associated with cigarette smoking are correlated with duration of smoking and degree of daily cigarette consumption, and cessation reduces an individual’s relative risks of tobacco‑related disease (1, 2). Thus, tobacco-related health risks are assumed to be due to repeated and sustained exposure to a range of smoke toxicants (3). Reducing the negative health impacts of tobacco use is a clear public health priority and has led to a series of regulatory and educational initiatives to persuade people not to smoke (4). Despite these efforts, smoking rates in adult populations worldwide remain at 15-25%. Although numbers are declining slowly in many countries (4), the World Health Organisation (WHO) has forecast that there will be around 1.5 billion tobacco smokers worldwide in 2050 (5). Current scientific study and public policy debate, therefore, are concerned with whether public health gains could arise from reducing future exposure to toxicants in people who continue to use tobacco through the development of new tobacco and nicotine products.

Smoke from conventional cigarettes is a complex and dynamic mixture of more than 5,600 identified chemical constituents (6), in both its particulate and vapour phases. Some of these chemicals have been identified as potential contributors to the harmful effects of cigarette smoke and can be evaluated by measuring the levels of these chemicals themselves, or their metabolites, in urine. Nicotine, a chemical also found naturally in tobacco leaf and which transfers into cigarette smoke, is primarily responsible for the addictive properties of cigarette smoking. Nicotine is rapidly absorbed into the bloodstream during cigarette smoking (7), from where it is rapidly distributed causing both systemic and central effects. In the central nervous system, nicotine acts at neuronal nicotinic receptors and this interaction may underpin its effects on mood and relaxation. The pharmacokinetic (PK) profile of nicotine during cigarette smoking is a rapid rise and fall in plasma nicotine concentrations. Correspondingly, the delivery of nicotine to the brain, and the consequent pleasurable effects experienced by the smoker, are also rapid.

**1.2 ELECTRONIC CIGARETTES (E-CIGARETTES)**

In the absence of both tobacco and combustion as a means of transferring nicotine into the inhaled matter, e-cigarettes deliver a vapour which is considered to contain significantly less chemical toxicants compared to cigarette smoke (8,9). Indeed, an independent scientific expert panel utilized a multi-criteria decision analysis approach, incorporating aspects of harm to users, to demonstrate the potential reduction in harm of e-cigarettes compared to combustible cigarettes (10), a conclusion recently endorsed by both Public Health England and the U.K. Royal College of Physicians (11-13). The use of e-cigarettes in helping smokers either reduce or quit smoking has been proposed as having the potential to play a major role in tobacco harm reduction (9, 11, 13), and this potential is further supported by data from large cross-sectional and longitudinal survey studies in the U.K. (14, 15). The cross-sectional data also suggest that e‑cigarettes are a more effective aid to smoking cessation than more traditional NRT products (14).

**1.2.1 VYPE EPEN E-CIGARETTES**

Vype ePen electronic cigarettes (e-cigarette) are sold by Nicoventures Trading Limited, a wholly-owned subsidiary within the British American Tobacco group of companies. Vype ePen consists of a reusable section (containing a rechargeable battery and an actuation button), a mouthpiece cover, and disposable cartridges (Supplementary Figure 2). EPen 2 is commercially-available and is currently marketed in the UK, with the cartridges being marketed as Vype ePen caps. EPen 3 which recently launched in the UK is a new update to the Vype ePen 2 e-cigarette.

**B.**


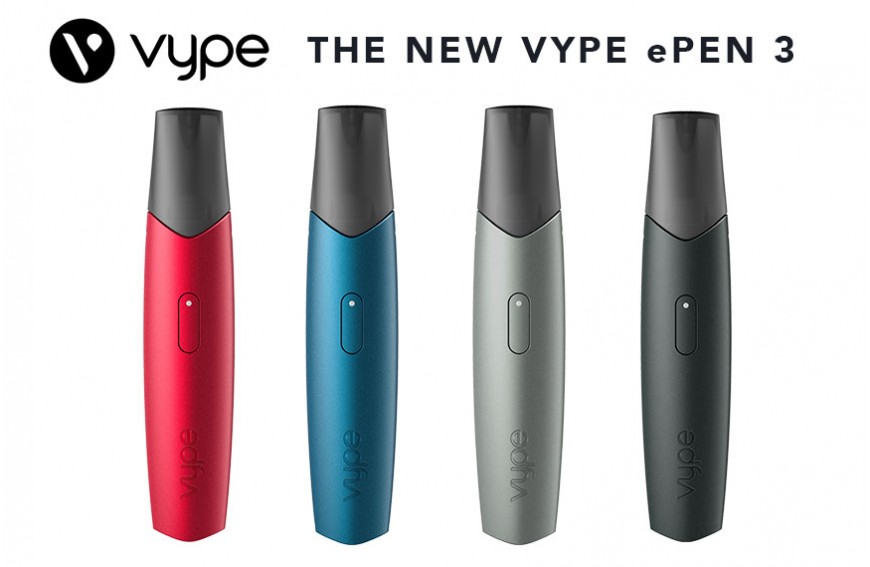

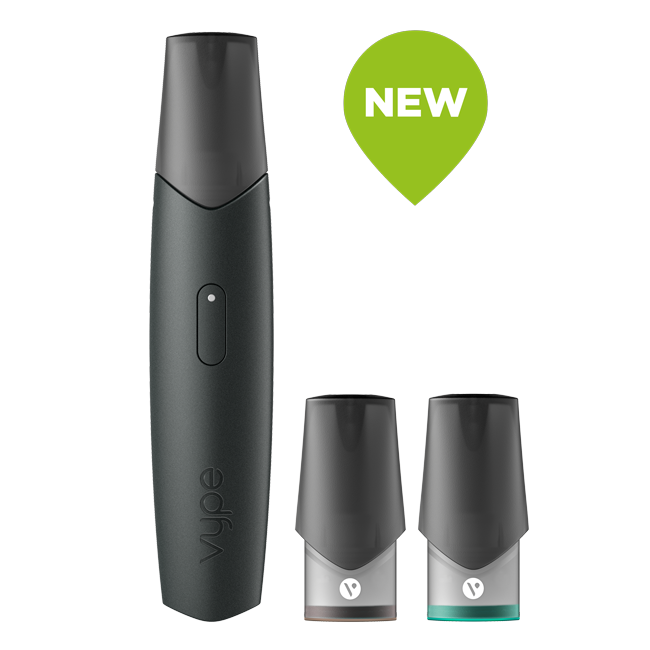


Removable mouthpiece

Activation button

Device electronics & battery


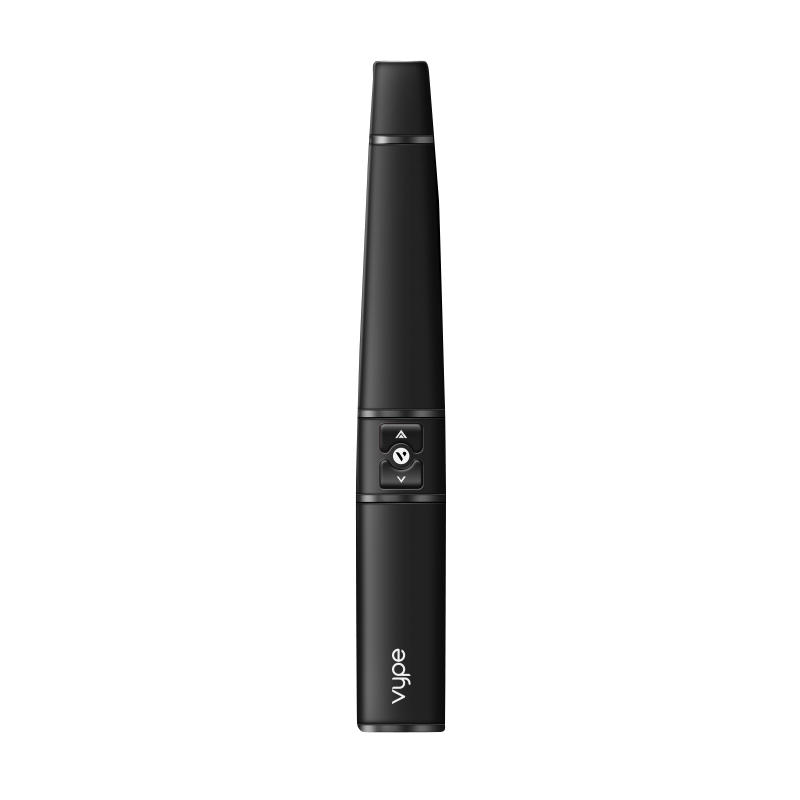

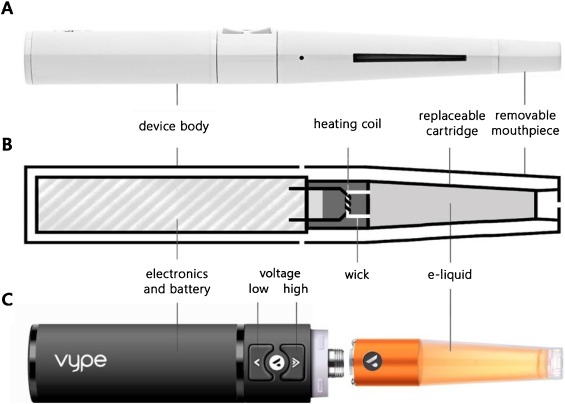


**A.**

**A.**

**Supplementary Figure 2:** Schematic representation of the **a)** Vype ePen 2 and **b)** Vype ePen 3 e-cigarette

The reusable section of both Vype ePen 2 and 3 contains a rechargeable 650 mAh battery which, when fully charged, will last a whole day under normal usage. The e-cigarette uses electronics to normalise the battery output irrespective of the battery charge state and ensure a consistent delivery over the charge/discharge cycle of the device.

The Vype ePen 2 cartridge is attached to the reusable section via a screw thread. Each Vype ePen 2 cartridge contains 1.58 mL (Supplementary Figure 2A) of e-liquid. The Vype ePen 3 cartridge is attached to the reusable section via a push fit engaging the plastic clips (Supplementary Figure 2B). Each Vype ePen 3 cartridge contains 2.0 mL of e-liquid. The e-liquid is held within a polypropylene tube within the cartridge, which is sealed using a semi-permeable ceramic disc. The ceramic disc transfers e-liquid to a wick which contains a small section surrounded by a heating coil. The heating coil is connected to terminals that connects it to the reusable section.

Vype ePen 2 and ePen 3 cartridges are designed to only be used with their respective e-cigarette devices. Cartridges are not refillable, and the e-liquid cannot be extracted from the cartridge without using considerable force to break it open. Vype ePen 3 come with a single 6 W power, while Vype ePen 2 comes with two power settings (4.4 W high power and 2.8 W low power).

**1.2.2 PURPOSE OF STUDY**

The purpose of this study is to characterise the pharmacokinetic profile of nicotine during a brief *ad libitum* or fixed use period of different variants of the e‑cigarette (see Supplementary Figure 2). Nicotine pharmacokinetics will be compared to those seen during smoking of a single conventional cigarette.

# **2. STUDY OBJECTIVES**

**2.1 PRIMARY OBJECTIVES**

- To determine kinetics of nicotine absorption into the blood of subjects using different variants of e-cigarettes and a cigarette.
- To compare nicotine delivery from different e-cigarettes to one another and with that from a conventional combustible cigarette.
- Compare the effect of *ad libitum* puffing versus fixed puffing regime.

**Secondary Objectives**

- Monitor heart rate during product use
- Evaluate product satisfaction with different e-cigarettes and a conventional cigarette
- Assess product usage
- Monitor safety profile of subjects using the study products

**2.2 STUDY ENDPOINTS**

**2.2.1 Primary Endpoints**

- Plasma nicotine C_max_, T_max_ and AUC_0-120_

**2.2.2 Secondary Endpoints**

- Heart rate
- Product satisfaction scores
- Product Usage (Mass loss from e-cigarette following use, calculated via cartridge weight pre and post puffing, puff count, during e-cigarette use)

**2.2.3 Other measures**

- Mouth level exposure (MLE) during cigarette use.

**2.2.4 Safety Endpoints**

- Medical history of study subjects
- Physical examination
- Vital signs
- Adverse events (AE)/serious adverse events (SAE) recording

**3. INVESTIGATION PLAN**

**3.1 STUDY DESIGN**

The study will be a single-centre, randomised, open-label, eight arm crossover study conducted in 24 healthy male and female volunteer subjects, who are daily e-cigarette users and active smokers (Maximum of 21 cigarettes per week) of combustible cigarettes and/or roll-your own cigarettes of >6 ISO tar cigarette per day. Subjects will be aged 19-60 inclusive and judged to be healthy by pre-study screening. Subjects must have been smoking regularly for at least one year and must not be trying to quit or planning on quitting. Product use status will be confirmed with a urinary cotinine level of ≥200 ng/ml (determined using One Step cotinine test kit) at screening. The study will be carried out at a single site where 24 subjects will be enrolled with 22 required to complete.

Potential subjects will attend the clinic for a screening visit. Subjects will be selected for enrolment into the study itself based on medical history, smoking status/history (tobacco use history questionnaire), physical examination, vital signs, serology, drugs of abuse and alcohol consumption screening. Pregnancy testing (serum at screening, urine at Day -1 and discharge) and FSH (screening only) will be performed on female subjects. During this visit, informed consent will take place and the subjects’ eligibility for inclusion will be determined. Smoking and vaping history will be assessed using questionnaires. Blood biochemistry, haematology, urinalysis^^[[6]](#footnote-6)^^, and urinary drugs of abuse screening will be performed. This screening visit will be performed no more than 28 days in advance of admission.

At admission (Day -1), the following assessments will be carried out: Urinary cotinine^^[[7]](#footnote-7)^^, Vital signs, symptom driven physical exam (if required), drugs of abuse and urine alcohol, urine pregnancy (females only). Upon completion of admission eligibility re-assessment, subjects will be allowed to familiarise themselves with the e-cigarette or cigarette, depending on their randomisation scheme.

On the morning of Day 1 after 12-hour abstinence from nicotine, subjects will be asked to smoke a single cigarette or use an e-cigarette, taking *ad libitum* or fixed puffs (10 puffs in total, one every 30 seconds) for a period of 5 minutes. If the subject finishes the cigarette before the 5-minute period is over, then this is the end of the smoking period. If this occurs, this will be captured in the CRF. Before, during and after the 5-minute smoking/vaping period, blood samples will be taken at specific time points for plasma nicotine analysis.

The same procedure described for Day 1 will be used for days 2 to 8 until all subjects have used all the investigational products. During each product use/blood sampling period, subjects will be asked to complete a product satisfaction questionnaire for the product that they used on that occasion and heart rate will be measured at intervals during the PK session.

Previous e-cigarette studies have shown that the absorption of inhaled nicotine is rapid. In order to obtain an accurate estimate of C_max_, it will be necessary to draw frequent blood samples during and following nicotine product use. Blood samples for nicotine pharmacokinetic analysis will be drawn before product use (-5 minutes) and at the following times after the first puff: 1, 3, 5, 7, 9, 15, 30, 45, 60, 90, and 120 minutes (a total of twelve 2.7 mL samples; total volume 33 mL). Hence, the total volume of all blood samples taken for pharmacokinetic analysis over the investigational period of the study will be approximately 300 mL.

During each study day, after the PK sessions, subjects will be allowed to use their own brand cigarette or e-cigarette for 2 hours, after which they will be asked to familiarise with the investigational product that would be used in the following day’s PK session, for a duration of 7 hours. After this period, subjects will abstain from smoking or vaping for a minimum of 12 hours.

Adverse event data and concomitant medication usage will be recorded throughout the study. A post-study follow-up will be conducted within 5 to 7 days after discharge. Assessments will follow-up on any adverse events experienced and concomitant medication administered. This will be achieved by a telephone call to each subject. A visit may be scheduled by the PI or designated individual.

Subjects in this study will be a minimum of 19 years of age. This is based on:

- The legal age to obtain nicotine/tobacco products in the UK is 18 years, plus an additional 1 year.
- Subjects will be required to have a vaping and smoking history of at least 1 year.

**3.2 BLINDING, RANDOMISATION AND SUBJECT IDENTIFICATION**

It is not possible to blind the test products. Each subject will be assigned a screening number beginning with 001 at the time of registering for the study.

Upon admission (Day -1), eligible subjects will be numbered sequentially from 001 (i.e. 001, 002 etc.). Replacement subjects will be assigned the same randomisation as the subject they are replacing, however, 100 will be added to the number (i.e., 101 would replace 001 etc.). The randomisation will determine the order in which the subjects will test the study e-cigarettes/cigarette at study days 1-8. The randomisation scheme for study products will be produced using a computer-generated pseudo-random permutation procedure using SAS version 9.3. A randomisation code for 24 subjects will be produced by Simbec and approved by the Sponsor.

A subject enrolment log and confidential subject identification list will be held in the Investigator Site File (ISF). Once assigned, screening and Randomisation numbers will not be re‑used.

**3.3 DETERMINATION OF SAMPLE SIZE**

From previous study undertaken, it has been observed that Cmax LS Means of smoking subjects can reach values around 13 -14 ng/mL in plasma and CVs between 40-60% among the different arms. Based on these data, a sample size calculation was performed using PROC POWER (SAS 9.4) to assess superiority between ePen3 using an 18 mg/mL e-liquid and no benzoic acid and the same product and liquid but containing medium levels of benzoic acid. This comparison assumes superiority to have a ratio between the C_max_ > 1 with a ratio between means of 1.4, β=0.2 and α=0.05. Based on these assumptions, 22 subjects completing the study would be the minimum required to successfully demonstrate superiority, providing an actual power of 0.814.

**4. STUDY POPULATION**

The study population will be made up of 24 male or female adult subjects who are current daily e-cigarette users and active smokers (Maximum of 21 cigarettes per week) of combustible cigarettes and/or roll your own cigarettes of >6 International Organization for Standardisation (ISO) tar cigarette per day (urinary cotinine level of ≥200 ng/mL at screening, reconfirmed at admission). The subjects will be selected from a large panel who have offered their services as volunteers to Simbec Research Limited for the purpose of undertaking REC‑approved studies on product safety, absorption and disposition. Geotargeting adverting campaign will be conducted using ethical approved adverts to gain interest of potential eligible volunteers.

This clinical trial can fulfil its objectives only if appropriate subjects are enrolled. The following eligibility criteria are designed to select subjects who are considered appropriate. All relevant medical conditions should be taken into consideration when deciding whether this protocol is suitable for a particular subject.

**4.1 INCLUSION CRITERIA**

**To be confirmed at Screening Visits:**

1. Subjects will be:
   1. males or females
   2. 19 to 60 years of age, inclusive, demonstrated by government issued proof of identification.
2. Subjects will have a:
   1. body mass index (BMI) of 18.5 to 30.0 kg/m^2^, inclusive
   2. body weight exceeding 52 kg (males) or 45 kg (females)
3. Subjects will be in good health, as judged by the PI or the appropriately qualified designee based on:
   1. medical history (confirmed by volunteer)
   2. physical examination
   3. vital signs assessment
   4. 12-lead ECG
   5. clinical laboratory evaluations
   6. lung function tests (During the screening period Day-28 to Day -1)
4. Subjects will have given their written informed consent to participate in the study and will have agreed to abide by the study restrictions.
5. Subjects must demonstrate the ability to comprehend the informed consent form (ICF), be able to communicate well with the PI or the appropriately qualified designee, understand and comply with the requirements of the study, and be judged suitable for the study in the opinion of the PI or the appropriately qualified designee.
6. Subjects will be willing to refrain from consuming alcohol within 24 hours prior to Admission.
7. Prior to study start, subjects must be current daily users of e-cigarettes and current smokers of conventional factory-made cigarettes and/or roll your own cigarettes, and must have done so for at least 1 year. Product use status will be confirmed with a urinary cotinine level of ≥200 ng/ml and product use history questionnaire at screening. Subjects must be smoking a maximum of 21 per week of >6 mg ISO tar cigarettes.

1. E-Cigarette users e-liquid must contain at least 18 mg/mL nicotine
2. Subjects will be willing to use the study products (cigarette product or e‑cigarette) and use only the products provided to them during clinical confinement, and to abstain from product use when instructed.
3. Women of non-childbearing potential may be included if they are either surgically sterile (hysterectomy and/or oophorectomy) or postmenopausal for more than 1 year and must have a negative pregnancy test result during screening. Women who are surgically sterile must provide documentation of the procedure by an operative report.

**4.2 EXCLUSION CRITERIA**

**To be confirmed at Screening Visit:**

1. Male subjects who do not agree, or whose partners of childbearing potential do not agree, to use a barrier method of contraception (i.e., a condom with spermicide) in addition to a second highly effective method of contraception used by their female partners or to refrain from donating sperm from Admission

(Day-1) until end of follow-up period.

1. Female subjects of childbearing potential who do not agree to use a highly effective method of birth control in conjunction with male barrier method contraception (i.e., a condom with spermicide) from the time of signing the ICF until end of follow-up period.
2. Female subjects who are pregnant or breastfeeding. This will be confirmed at Screening and Admission. Any female subject who becomes pregnant during this study will be withdrawn.
3. Subjects who have donated:

- ≥400 mL of blood within 90 days prior to screening
- plasma in the 7 days prior to screening
- platelets in the 6 weeks prior to screening

1. Subjects who have an acute illness (e.g. upper respiratory tract infection, viral infection, etc.) requiring treatment within 4 weeks prior to screening.
2. Subjects who are self-reported non-inhalers (smokers/vapers who draw smoke/aerosol from the cigarette/e-cigarette into the mouth and throat but who do not inhale). Subjects who are observed as non-inhalers at Admission by the clinic staff will be excluded.
3. Subjects who, prior to enrolment, are planning to quit smoking/vaping in the next 12 months. All subjects will be informed that they are free to quit smoking/vaping and withdraw from the study at any time.
4. Subjects who have a significant history of alcoholism or drug/chemical abuse within 24 months prior to Screening, as determined by the PI or the appropriately qualified designee.
5. Subjects who have a positive urine drugs of abuse or alcohol screen (confirmed by repeat) at Screening or Admission
6. Subjects who:

- have serum hepatitis
- are carriers of the hepatitis B surface antigen (HBsAg)
- are carriers of the hepatitis C antibody
- have a positive result for the test for human immunodeficiency virus (HIV) antibodies.

1. Subjects who have used prescription or over-the-counter (OTC) bronchodilator medication (e.g. inhaled or oral β-adrenergic agonists) to treat a chronic condition within the 12 months prior to screening.
2. Subjects who have received any medications or substances (other than tobacco) which are known to be strong inducers or inhibitors of cytochrome P450 (CYP) enzymes within 14 days or 5 half-lives of the drug (whichever is longer) prior to screening.
3. Subjects who perform strenuous physical activity (exceeding the subject’s normal activity levels) within 7 days prior to Screening or Admission.
4. Subjects who are unable to communicate effectively with the PI/study staff (i.e. language problem, poor mental development, or impaired cerebral function).
5. Subjects who are unwilling or unable to comply with the study requirements.
6. Employees and immediate relatives of the tobacco industry or the clinical site.
7. Participation in a new chemical entity clinical study within the previous 3 months or a marketed drug clinical study within the 30 days before first dose of IMP.
8. Subjects who have any clinically relevant abnormal findings on the physical examination, medical history, ECG, lung function tests (During the dcreening period Day-28 to Day -1) or clinical laboratory panel, unless deemed not clinically significant by the PI or the appropriately qualified designee.
9. Subjects who do not use a flavoured e-cigarette e-liquid
10. Subjects who have been diagnosed with a significant history of urticaria or asthma (childhood asthmas is acceptable).
11. Subjects who have, or who have a history of, any clinically significant neurological, gastrointestinal, renal (including urinary tract infection or nephrolithiasis), hepatic, cardiovascular, psychiatric, respiratory, metabolic, endocrine, haematological or other major disorder that, in the opinion of the PI or the appropriately qualified designee, would jeopardise the safety of the subject or impact on the validity of the study results.
12. Subjects who have had an acute illness (e.g. upper respiratory tract infection, viral infection, etc.) requiring treatment within 4 weeks prior to screening.
13. Subjects who have had any treatment with smoking cessation medications (e.g. Bupropion,Varenicline or any NRTs) within 30 days of the planned first product use occasion.
14. Subjects who have previously been diagnosed with any form of malignancy.
15. Subjects who have previously randomised into or withdrawn from this study.
16. Subjects who, in the opinion of the PI or the appropriately qualified designee, should not participate in this study.

**4.3 SUBJECT WITHDRAWAL**

Any subject may discontinue from the study at any time and for any reason, either at the discretion of the Investigator, the Sponsor or at the request of the subject. The reason for such a premature discontinuation must be clearly documented in the CRF.

The Investigator may withdraw a subject from the study at any time if he considers that the subject’s health is compromised by remaining in the study or the subject is not sufficiently cooperative.

Subjects may be withdrawn from the study prematurely for the following reasons:

1. If a subject experiences an intolerable AE, premature discontinuation will be at the discretion of either the Principal Investigator (PI, or the appropriately qualified designee) or the subject, independent of the relationship of the AE to the study test product. The appropriate AE case report form (CRF) page must be completed.
2. If a subject develops a non-fulfilment of inclusion/exclusion criteria or concurrent disease, which at the discretion of the PI (or the appropriately qualified designee), no longer permits the subject’s participation in this study.
3. If certain deviations occur during the conduct of the study. This may include the subject’s erroneous inclusion in the study. Any protocol deviations detected during the study should be corrected when possible and the subject should be allowed to continue if considered appropriate at the discretion of the PI. All protocol deviations will be fully documented and considered for their effect on study objectives. Deviations that should lead to subject discontinuation from the study include:

- deviations which could affect subject’s safety (e.g. illness requiring treatment[s]) which in the clinical judgement of the PI (or the appropriately qualified designee, or after discussion with the sponsor’s medical contact) might invalidate the study by or the willingness of the subject to comply with the study activities.

1. If the subject is uncooperative, including non-attendance. In these cases, efforts should be made by the clinic personnel to ascertain the reason and to ensure subject’s attendance as soon as possible.
2. Subject’s personal request: the subject could decide, at any moment of the study, to stop his/her participation. The PI, or the appropriately qualified designee, should ensure this is not due to AEs, in which case, this reason should be selected. Subjects do not have to provide a reason for withdrawing from the study if they do not wish to do so (reason for withdrawal should be recorded as ‘not given’).
3. Pregnancy.
4. Premature cancellation of the study.

If a subject discontinues the study prematurely, the following should be done:

1. Complete the Final Evaluation CRF page, indicating the date of the last test product administration along with the reason for premature discontinuation.
2. Complete all CRF pages as appropriate up to the date of the premature discontinuation, whatever the reason for withdrawal may be (if given).

**4.3.1 Safety Follow‑up**

Subjects who, after randomisation, discontinue the trial prematurely should be encouraged to participate in the same follow-up procedure as those who have completed the study. This will be done by a telephone call to the subjects as soon as possible after their withdrawal from the study and the corresponding CRF pages should be completed. Symptom-driven vital signs and physical examination may also be performed if necessary.

If their withdrawal is due to safety evaluations, then the subjects should be followed up until these return to baseline levels or until the Investigator has determined that these events are no longer clinically significant.

Subjects who develop an adverse event at any time during the study will be followed up until any required evaluations have returned to baseline or until the Principal Investigator has determined that these events are no longer clinically significant.

Reported adverse events will be followed until resolution whenever possible.

**4.3.2 Subject Replacement**

Subjects prematurely withdrawn from study irrespective of the time of withdrawal and completed sessions will be replaced in order to reach the pre-planned target number of evaluable subjects.

**4.4 ADDITIONAL ADVICE FOR STUDY POPULATION**

**Alcohol Intake**

Alcohol should be avoided completely for a period of 24h prior to screening and admission. Any deviation outside this alcohol intake restriction will be assessed on a case by case basis at the Investigator’s and Sponsor’s discretion (provided the subject’s alcohol intake will not impact the safety aspects and objectives of the study).

**Poppy Seeds**

Subjects will be advised that they must not eat food containing poppy seeds for 3 days before screening and admission on day -1, as consumption of poppy seeds can lead to a positive opiate result in the drugs of abuse test.

**Exercise**

Subjects will be advised that they should avoid unusually intense or strenuous exercise during the study, in the period starting from screening visit and before admission.

**Blood Donation**

Subjects will be advised that they should not donate blood for at least three months after discharge.

**Food Intake**

Subjects will be asked to abstain from food for a period of four hour prior to the screening visit.

Breakfast will be served pre dose to allow a fasting window of 1 hour fast prior to dosing.

Lunch will be served: 4 h post-dose.

Dinner will be served: 8 h post-dose.

Snack will be served: 12 h post-dose.

On all non-dosing study days, whilst resident in the Clinical Unit, meals will be served at standard times.

Subjects will choose meals from a standard menu while resident at the Clinical Unit.

**5. TEST PRODUCTS**

**5.1 DESCRIPTION, IDENTIFICATION AND STORAGE**

All study products will be supplied by the Sponsor, along with appropriate documentation. Study products will be kept in their packages until administration and stored at room temperature. All products will be securely stored at the investigational site, in an area which is locked and with restricted access to only those directly involved in the study. No special procedures are required for the safe handling of the products and the study product must not leave the clinic site. Subjects should be informed that, in the unlikely event of an e-cigarette cartridge leaking they should wash the affected area with copious amounts of cold water, remove any contaminated clothing, and inform the site staff immediately.

The cigarettes and EPEN2.0BT18 will be procured off the shelf through commercial retail, while all ePen 3 (EPEN3.0BT18, EPEN3.0MB18VP, EPEN3.0MB12VP and EPEN3.0MB30VP) formulations would be provided by the Sponsor specifically for the purpose of the study

**5.2 PACKAGING AND LABELLING OF TEST PRODUCTS**

The containers housing the cigarette and e-cigarette products will be labelled

**5.3 TEST PRODUCT ADMINISTRATION**

Test products will be administered on admission (Day -1 familiarisation) and on the morning of each visit (Visits 1 to 8). Only one test product will be administered per day. The procedures for the preparation, dispensing and collection of each product will be detailed in the Study Operational Manual.

**Cigarette**

Subjects will be asked to smoke a single Benson & Hedges Skyblue cigarette (ISO^^[[8]](#footnote-8)^^ yield 7 mg/cig nicotine) by taking *ad libitum* or fixed puffs (10 puffs, one every 30 seconds) over a period of 5 minutes. If the cigarette remains lit at the end of the 5-minute period, no further puffing will be allowed. If the subject reaches the end of their cigarette before 5 minutes have elapsed, then this marks the end of the smoking period. The number of puffs taken will be counted by clinic staff and recorded in the CRF. Cigarette butts will be collected after the cigarette smoking PK session.

**E-cigarette**

Subjects will be asked to use one e-cigarette by taking *ad libitum* or fixed puffs (10 puffs, one every 30 seconds) over a period of 5 minutes. Subjects will only be allowed to use their randomised e-cigarette during the PK session and will not be allowed to use a different product or smoke any type of regular cigarette. The number of puffs taken will be counted by clinic staff and recorded in the CRF.

**5.4 PRODUCT USE COMPLIANCE**

To ensure product use compliance in the clinic, all products will be administered under the supervision of suitably qualified staff. All subjects will receive training by clinic staff on how to operate the different e-cigarettes.

**5.5 TEST PRODUCT ACCOUNTABILITY**

Records will be maintained showing the receipt and disposition of the study supplies. The Sponsor will be permitted, at intervals, and upon request during the study, to check the supplies storage and dispensing procedures and records.

Samples of the test products will be retained by the Sponsor for 1 year after completion of the study. Following completion of the clinical phase of the study, and Sponsor review of accountability, all e-cigarette devices, charging cables, and used and unused supplies will either be returned to the Sponsor, together with accountability records, or will be destroyed and Certificates of Destruction provided to the Sponsor. The Study CRA will review all product accountability records for accuracy and completeness.

**5.6 CONCOMITANT MEDICATION**

Medications considered necessary for the welfare of the subject, and which are not expected to interfere with the evaluation of study treatments, may be taken at the discretion of the Investigator.

**5.6.1** **Prior and Concomitant Medications and Therapies**

Subjects will not take any prescription or over-the-counter medication (including herbal medications) during the study until completion and discharge, unless the Principal Investigator has given their prior consent. For female subjects, hormonal contraceptives are acceptable. For all subjects, painkillers (paracetamol) are permitted. Subjects must be made aware that they must inform the Investigator before taking any new treatment during their participation in the trial.

If any medication is required, the name, strength, frequency of dosing and reason for its use will be documented in the subject's CRF.

**5.6.2** **Prohibited Medications**

Subjects should not use any drugs or substances (except tobacco) known to be strong inducers or inhibitors of CYP enzymes (formerly known as cytochrome P450 enzymes) within 14 days prior to first product use. For a list of such drugs and substances, please refer to http://medicine.iupui.edu/clinpharm/ddis/main-table/.

**6. STUDY PROCEDURES**

**6.1 STUDY PROCEDURES BY VISIT**

**6.1.1** **Screening Visit**

Subjects will undergo screening assessments no more than 28 days in advance of the first product administration. Enrolment in the study is defined as the signing of the informed consent. All subjects enrolled in the study will be listed on a subject enrolment log. It is anticipated that recruitment of subjects for the study will begin in October 2018.

The following information and procedures will be recorded and performed as part of the screening assessments:

- Informed consent
- Adverse event recording
- Inclusion and exclusion criteria
- Complete medical history
- Demography (including gender, ethnic origin, age, height, weight and BMI)
- Prior and concomitant medication
- Vital signs (seated blood pressure and pulse rate, body temperature)
- 12-lead ECG
- Lung function testing (During the screening period Day-28 to Day -1)
- Physical examination by the study physician
- Urine drug of abuse including urine alcohol screen
- Serum pregnancy test and FSH for female subjects
- Tobacco and e-cigarette history and habits, including urinary cotinine screen, Fagerstrom Test for Nicotine Dependence, and tobacco/e-cigarette use history questionnaires.^^[[9]](#footnote-9)^^
- Current brand of cigarettes smoked. Subjects should bring their e-cigarette device and preferred e-liquid. (if available a pack of their cigarettes to the screening visit so that a photo/photocopy of this can be taken)
- Biochemistry, haematology, serology and urinalysis.^^[[10]](#footnote-10)^^

**6.1.2 Admission Day -1**

Admission day -1 will be held within 28 days after the Screening Visit. On admission the following assessments will be carried out:

- Inclusion/exclusion criteria
- Urinary cotinine screen
- Medical history
- Adverse event recording
- Vital signs
- Physical examination (Symptom-driven physical examination is required)
- Urine drugs of abuse and alcohol screen
- Pregnancy test (urine)
- Concomitant medication
- Product Usage (Use count, puff count, cartridge weight and MLE)
- Heart (pulse) rate
- Lung function testing (if not performed at Screening visit)

Subjects will be re-assessed for eligibility and asked to familiarise with the combustible cigarette or e-cigarette product they will use the following day for 7 hours. After familiarisation, subjects will abstain from smoking/vaping for 12 hours. Subjects will remain in the clinic throughout the study until discharge on day 8.

**6.1.3 Clinic Phase (Day 1-8)**

During clinic stay days 1-8, subjects will use their randomly-assigned e-cigarette for pharmacokinetic and product satisfaction analysis. During these study days, subjects will undergo their PK session for 2 hours, following this PK session subjects will be allocated 2 hours where they can use their own brand products, the next 7-hours will be allocated for study product familiarisation. After the familiarisation period, subjects will undergo a 12-hour overnight nicotine abstinence. This process will repeat until each subject has used all study products. At the beginning and end of each e-cigarette use, the e-cigarette cartridge will be weighed. The values will be subtracted from one another to determine the loss of mass from the cartridge during the puffing period.

Cigarette butts will be collected after each use for mouth level exposure (MLE) analysis. During both PK and familiarisation sessions with the EPEN2.0BT18, subjects are to use the device in high power (4.4 W) at all times.

Prior to discharge from the clinical unit the following assessment will be carried out:

- Adverse event recording
- Vital signs
- Symptom driven physical examination if required
- Urinary pregnancy test (females only)
- Concomitant medication recording

**6.1.4 Follow-up**

The Investigator will obtain information on any new adverse events and new/changes to concomitant medication since the last product use. Provided there are no adverse events which require further attention, the subject’s participation in the study will be complete. This will be performed by a phone call to the subjects with a visit scheduled only if necessary. If a visit is necessary, vital signs will be taken and a symptom-driven physical examination performed.

**6.1.5 Pharmacokinetic and Satisfaction Assessments**

Full details of all procedures, criteria and equipment relating to the pharmacokinetic and satisfaction assessments will be provided in the Study Operational Manual. Training will be provided where appropriate.

**6.2 BLOOD SAMPLING FOR THE ANALYSIS OF NICOTINE**

Blood samples (2.7 mL) will be taken either by direct venepuncture or from a cannula placed in a forearm vein, at the following times: -5, 1, 3, 5, 7, 9, 15, 30, 45, 60, 90 and 120 minutes. Blood samples will be collected into a lithium heparin monovette tube.

No later than 60 minutes after collection, samples will be centrifuged at 3500 RPM and at 4°C for 10 minutes. The plasma will be transferred to 2 polypropylene screw cap tubes and stored frozen at -20°C (tolerance +10°C) or below within 120 minutes from collection.

**6.2.1 Nicotine bioanalysis**

Plasma samples will be analysed for nicotine at Seirian Laboratories using a validated liquid chromatography-tandem mass spectrometry (LC-MS/MS) method according to applicable local SOPs. All samples will be stored for up to one years and then destroyed.

**6.3 SAFETY ASSESSMENTS**

**6.3.1 Adverse Events**

The condition of each subject will be monitored throughout the study from the Screening Visit until the follow-up contact. Any adverse events and remedial action will be recorded in the subject’s CRF. The nature, time of onset, duration and severity will be documented, together with an Investigator’s opinion of the relationship to product administration.

Adverse event definitions, assignment of severity and causality, and procedures for reporting serious adverse events are defined in Section 11.

**6.3.2 Vital Signs, Height, Weight and BMI**

Seated (after the subject has been seated for at least 5 minutes) blood pressure and pulse rate, and body temperature, will be measured. If there is any suspicion of unreliable measurement, blood pressure, pulse and/or body temperature will be measured again. The value obtained this time will be considered as definitive and should be properly recorded on the CRF page.

Height in metres (to the nearest cm) and weight in kilograms (to the nearest 0.1 kilogram) in light indoor clothing and without shoes will be measured.

BMI = body weight (kg) / [height (m)]^2^ will be calculated.

**6.3.3 Physical Examination and health screens**

A physical examination will be performed at the Screening Visit and discharge (if required) according to normal procedures performed routinely at the investigational site. The examination will include: ears, nose and throat, ophthalmological, dermatological, cardiovascular, respiratory, gastro-intestinal, central nervous system, lymph nodes, musculo-skeletal. Other body systems can be examined if required, at the discretion of the Principal Investigator.

Blood samples will be obtained for biochemistry, haematology and serology analysis. A urine sample will be obtained for urinalysis^^[[11]](#footnote-11)^^ and drugs of abuse (including cotinine and alcohol). A 12-lead ECG will also be performed.

**6.3.4 Smoking and nicotine use tests**

Subjects will also be asked to provide a urine sample for a cotinine screen and they will only be enrolled into the study if their urine cotinine level is greater or equal to 200 ng/mL

**6.3.5 Pregnancy Test**

To be entered into and to complete the study, female subjects must have a negative serum pregnancy test at screening, urine pregnancy at admission (day -1) and on discharge.

**6.3.6 Urine Drugs of Abuse Screen**

Subjects will be asked to provide urine samples for a drugs of abuse screen at the Screening Visit and admission on Day -1. Urine samples will be screened for the presence of the following drugs of abuse: Amphetamine, Barbiturate, Benzodiazepine, Cannabinoid (for tetrahydrocannabinol), Cocaine, Methadone and Opiate (for Morphine).

**6.3.7** **Total Blood Volume**

During the study itself, approximately 300 mL of blood will be withdrawn from each subject. An additional 15 ml will be required for biochemistry and haematology during screening.

**7. DATA ANALYSIS**

A detailed Statistical Analysis Plan describing the full methodology to be used will be written and finalised prior to database lock. Data analysis and graphical output will be carried out using SAS version 9.3 or higher.

**7.1 Analysis Sets**

**Safety Set (Safety):** All randomised subjects who partake in at least one IP will be included in the Safety Set.

This analysis set will be used for baseline and safety summaries as well as for all study listings.

**PK Set (PK):** Subjects will be allocated to the PK Set on a per product basis. Subjects will be assigned to the PK Set for an IP where they have received the specific IP, have sufficient plasma nicotine concentration-time profiles and do not violate the protocol (major protocol violation) in a way that may invalidate or bias the results.

**7.1 DEMOGRAPHY AND BASELINE CHARACTERISTICs**

All demographic and baseline data will be listed.

**Disposition**: Subject disposition will be listed with any withdrawals flagged. Frequencies (number and %) of the total number of subjects dosed, completed and prematurely discontinued (including reason for discontinuation) from the study will be summarised. Additionally, the frequency of subjects within each analysis set will be summarised.

**Demographics**: Demographic data will be listed. Descriptive statistics (number of subjects in the analysis population (N), number of subjects with non‑missing observations (n), mean, standard deviation (SD), minimum, median and maximum) will be tabulated by gender and overall for the continuous variables age, height, weight and BMI and frequencies (number and %) for the categorical variable race/ethnicity.

**Tobacco and e-cigarette history and habits**: Product use history and Fagerström Test for Cigarette Dependence index scores will be listed and summarised using frequencies (n, %).

**7.2 SAFETY AND TOLERABILITY DATA**

All safety data will be listed.

**Adverse Events:** All AEs will be coded according to the latest version of the Medical Dictionary for Regulatory Activities (MedDRA).

All AEs, including those which occurred prior to the first dose of IP, will be listed. Only treatment emergent adverse events (TEAEs), i.e., existing conditions that worsen or events that occur during the course of the study after administration of IP, will be included within the summary tables.

An overall summary of AEs will be produced including the number of TEAEs; the number and % of subjects reporting at least 1 TEAE, serious TEAE, TEAE leading to withdrawal from the study; the number and % of subjects reporting TEAEs by severity and relationship to IP.

The number of TEAEs and the number and % of subjects reporting at least 1 TEAE will be tabulated by system organ class (SOC) and preferred term. In addition, the number and % of subjects reporting TEAEs will be tabulated by maximum severity and strongest relationship to IMP.

**Heart Rate**: Heart rate data will be listed with any out of normal range values flagged. Descriptive statistics (N, n, mean, SD, minimum, median and maximum) of absolute and change from baseline (pre‑administration) values at each time point will be tabulated.

Physical examination data will be listed. Vital signs, 12-lead ECG and safety laboratory data (biochemistry, haematology, urinalysis) will be listed with any out of normal range values flagged. Laboratory test results which are out of normal range will also be presented separately along with normal reference ranges

**7.3 PHARMACOKINETIC DATA**

Individual plasma nicotine concentration‑time data will be listed and summarised for each product by gender and overall. Individual and mean concentration‑time data will also be plotted with and without 95% CI of the mean for each product on both linear and semi‑logarithmic scales.

The following pharmacokinetic (PK) parameters will, where possible, be derived from the plasma concentrations of nicotine, using non-compartmental procedures.

| Parameter | Definition |
| --- | --- |
| C_max_  T_max_  AUC_0-120_ | Maximum observed plasma concentration  Time of maximum observed plasma concentration  Area under the curve from time zero to the last sample collection (120 min) |

Additional PK parameters may be determined where appropriate.

Area under the curve (AUC_0-120_) will be calculated using the linear trapezoidal method when concentrations are increasing and the logarithmic trapezoidal method when concentrations are decreasing, known as the Linear Up-Log Down method. Where AUC_0-120_ is defined as the area under the curve from T = 0 sample collection time-point until the last collection at 120 min timepoint.

The derived PK endpoints will be summarised for each product by gender and overall, using the descriptive statistics N, n, mean, SD, coefficient of variation (CV%), minimum, median, maximum and geometric mean (with the exception of T_max_).

Statistical comparisons will performed in order to test the following hypotheses:

1. The addition of benzoic acid to the ePen 3 e-liquid results in a higher level of plasma nicotine compared to without benzoic acid.
2. Use of ePen 3 increases plasma nicotine to levels that are comparable to a combustible cigarette.
3. The plasma nicotine level is higher following ePen 3 use compared to ePen 2.
4. Higher concentrations of nicotine and benzoic acid in the ePen 3 e-liquid increases the level of plasma nicotine compared to lower concentrations.
5. A fixed puffing protocol yields a different plasma nicotine PK profile to that of an *ad libitium* puffing protocol.
6. T_max_ during *ad libitum* use of ePen 3 is no later then T_max_ during ad libitum use of ePen 2.

In order to account for residual plasma nicotine at baseline, the baseline value expressed as a percentage of C_max_ (baseline %C_max_) will be derived for each subject per product.

Following logarithmic transformation, C_max_ and AUC_0-120_ values will be subjected to an analysis of covariance (ANCOVA) including fixed effects for sequence, period and product and a random effect of subject nested within sequence, with baseline %C_max_ as a covariate. Point estimates and 95% confidence intervals (CI) will be constructed for the contrasts of interest between each of the products using the residual mean square error obtained from the ANOVA. The point and interval estimates will be back-transformed to give estimates of the ratios of the geometric least squares means (LS mean) and corresponding 95% CI. In addition, estimated geometric means and 95% CI will be presented for each product.

A comparison of T_max_ values obtained from ePen 3 during ad libitum use will be compared to those obtained from ePen 2 during ad libitum use will be performed using the Wilcoxon Signed Rank Test. In addition, the Hodges-Lehmann estimate of the median difference in T_max_ and 95% CI will be presented.

**7.4 OTHER DATA**

**Product Satisfaction Assessment:**

Product satisfaction data will be listed by subject and summarised for each product using frequencies (n, %).

**Cigarette Filter Analysis:**

The Sponsor has developed a methodology that estimates the level of exposure of smoke constituents in the smokers’ mouth (mouth level exposure (MLE)). This methodology (St Charles et al., 2009) involves collection of cigarette butts/filters post smoking. A portion is cut from the mouth end of each filter and these ‘tips’ are retained. Filter tips collected over the familiarisation period will be pooled, also single tips from the PK session will be collected separately and sent to Sponsor. The UV absorbance is related to the tar content of tips. These tip data are compared with calibration curves obtained by machine smoking cigarettes using a number of puffing regimes. The calibration curves are obtained by plotting smoke nicotine and tar yield against tip nicotine and tip UV absorbance respectively where the slopes of the curves represent the filtration efficiency of the cigarette filter. Therefore, analysis of the cigarette filter enables an estimate to be made of the amount of smoke that exited the filter and entered the smokers’ mouth (MLE).

Product usage data including mass difference from e-cigarette device pre and post puffing, puff count, and mouth level exposure (MLE) during cigarette use will be listed and summarised for each product using the descriptive statistics N, n, mean, SD, minimum, median and maximum.

**8. REPORTS AND PUBLICATIONS**

**8.1 REPORTS TO THE ETHICS COMMITTEE (EC)**

Completion of the study will be reported to the EC responsible for the study within 90 days of completion of the last subject’s final study procedures. In the event of the study being prematurely terminated a report will be submitted to the EC responsible for the study within 15 days of termination.

**8.2 CLINICAL STUDY REPORT**

The CRO will prepare a clinical study report (CSR). The report will be written in accordance with the International Conference on Harmonisation (ICH) Note for Guidance on Structure and Content of Clinical Study Reports.

The draft report may be submitted for Quality Assurance audit, the findings of which will be incorporated into the final version.

**8.3 PUBLICATIONS**

The publication policy for this study will be part of a separate agreement between the Investigator and the Sponsor.

**8.4 CLINICAL STUDY REGISTRATION**

This study will be registered on HRA website (within 6 weeks of first subject first visit).

**9. ETHICAL CONSIDERATIONS**

**9.1 INDEPENDENT RESEARCH COMMITTEE APPROVAL**

This study protocol will be submitted to an REC responsible for the study for review and approval. The approval of the responsible REC must be obtained before commencement of any study procedures.

All substantial protocol amendments must be approved by the REC responsible for the study. The REC will be informed of minor amendments to the protocol which do not require REC approval

If the study is stopped due to adverse events it will not be recommenced without reference to the REC responsible for the study.

The REC will be informed that the Investigator is a commercial organisation and that the study is funded by British American Tobacco (Investments) Limited as the Sponsor. The subjects who take part in the clinical trial will be compensated for their inconvenience and will be informed that there may be no benefits gained by their participation. All potential conflicts of interest will be declared by the Investigator.

The Principal Investigator will maintain records of all correspondence with the REC.

**9.2 INFORMED CONSENT**

Prior to the commencement of the study, each subject will be provided with a subject information sheet giving details of the test products, procedures, and potential risks involved. Subjects will also be instructed that they are free to obtain further information from the Investigator and that they are free to withdraw their consent and to discontinue their participation in the study at any time without giving a reason.

All subject names will be filed confidentially by the Investigator. Subjects will be identified in documentation and throughout evaluation by the number allotted to them during the study. The subjects will be told that all study findings will be stored electronically and handled in the strictest confidence.

Following discussion of the study with Investigator personnel at the study site, subjects will be invited to sign the consent form, in the presence of a physician, to indicate that they are freely giving their informed consent to participate in the study.

**9.3 DECLARATION OF HELSINKI**

This study will be conducted in accordance with the relevant articles of the Declaration of Helsinki as adopted by the 18th World Medical Assembly in 1964 and as revised in Tokyo (1975), Venice (1983), Hong Kong (1989), South Africa (1996) and Edinburgh (2000); Notes of Clarification added Washington (2002) Tokyo (2004), South Korea (2008) and Brazil (2013).

**9.4 STUDY TERMINATION**

If, in the opinion of the Investigator or their appropriately qualified designee, the clinical observations in the study suggest that it may be unwise to continue, part of or the entire study may be terminated after consultation with the Sponsor. In addition, the Sponsor may terminate part of or the entire study for safety or administrative reasons. A written statement fully documenting the reasons for study termination will be provided to the research ethics committee (REC).

**10.** **GOOD CLINICAL PRACTICE (GCP)**

The Principal Investigator shall be responsible for ensuring that the clinical study is performed in accordance with the ICH Harmonised Tripartite Guideline for Good Clinical Practice. The study shall also be conducted in accordance with GCP guildlines E6 (R2) and applicable local standard operating procedures (SOPs).

**10.1 SPONSOR’S OBLIGATIONS**

The Sponsor will provide an Investigators Brochure for the Vype ePens. The cigarette and e-cigarettes devices are commercially available products marketed in the UK. However, some of the e-liquids have been made specifically for this study and are not commercially available.

A designated professional Clinical Research Associate (CRA) contracted by the sponsor will conduct monitoring visits at suitable intervals throughout the study. These visits will be for the purposes of verifying adherence to the protocol and GCP, and that accurate and complete recording of data in the CRFs and test product inventory forms. A monitoring plan will be drawn up with the CRA and the Sponsor at least two weeks prior to study start and prior to the conduct of the Site Initiation Visit (SIV).

**10.2 INVESTIGATOR'S OBLIGATIONS**

Prior to initiation of this study, the Principal Investigator and Study Physician will approve this protocol by signing the approval signature page. This signature confirms that the study will be performed in compliance with this protocol.

**10.3 ADHERENCE TO THE PROTOCOL**

The Principal Investigator undertakes to adopt all reasonable measures to record data in accordance with this protocol. Under practical working conditions, however, some minor variations may occur due to circumstances beyond the control of the Investigator. All such deviations will be documented in the study records, together with the reason for their occurrence; where appropriate, deviations will be detailed in the clinical study report.

To ensure compliance with GCP and all applicable regulatory requirements and if necessary, a pre-study quality assurance audit will be conducted in agreement with the Sponsor.

**10.4 DATA QUALITY ASSURANCE**

In accordance with applicable regulations, GCP, and procedures, the study CRA will contact the site prior to the start of the study to review with the site staff the protocol, Case Report Forms (CRFs), study requirements, and their responsibilities to satisfy regulatory, ethical, and CRU/Sponsor requirements. During the course of the study the CRA will visit the CRU regularly to check the completeness of the subjects’ records (including the Volunteer (Subject) Master Files), the accuracy of entries into the CRFs, the adherence to this protocol and to ICH GCP, the progress of enrolment and also to ensure the storage, and handling and accountability of the study products. The Investigator and key study personnel will be available to assist the CRA during these visits. When reviewing data collection procedures, the discussion will also include identification, agreement and documentation of data items for which the CRF will serve as the source document.

The CRA will monitor the study consistent with the demands of the study and site activity to verify that the:

- Data are authentic, accurate, and complete.
- Safety and rights of subjects are being protected.
- The study is conducted in accordance with the currently approved protocol and any other study agreements, GCP, and all applicable regulatory requirements.

The Sponsor agrees to allow the CRA access to all relevant documents.

**10.5 SITE CLOSURE OR TERMINATION OF STUDY**

Upon completion or premature discontinuation of the study, the CRA will conduct site closure activities with the Sponsor and the Investigator or site staff, as appropriate, in accordance with applicable regulations such as GCP.

In addition, the Sponsor reserves the right to temporarily suspend or prematurely discontinue this study at any time for reasons including, but not limited to, safety or ethical issues or severe non-compliance.

The Principal Investigator will promptly inform the Sponsor if the study is suspended or prematurely discontinued for safety reasons. If required by applicable regulations, the Principal Investigator must also inform the REC promptly and provide the reason for the suspension or premature discontinuation.

**10.6 DATA STORAGE AND ARCHIVING**

All primary data generated by the study site, or copies thereof (e.g. laboratory records, CRFs, data sheets, correspondence, photographs and electronic records), which are a result of the original observations and activities of the clinical study, and are necessary for the reconstruction and evaluation of the study report, will be retained in the study site’s archive for a period of 10 years after issue of the final report.

**10.7 VISITS BY REGULATORY AUTHORITIES**

With the exception of statutory regulatory authority inspections, the study Sponsor expects to be consulted in the event of inspection of a study site by an outside authority before any inspectors are permitted access to any of the study records or the study areas.

**10.8 FINANCES AND INSURANCE**

The finances for this study will be the subject of a separate agreement between the Investigator and the Sponsor, British American Tobacco (Investments) Limited. The funding for this study will be provided by the Sponsor. The Sponsor will provide compensation where required by applicable law. The Sponsor holds Clinical Trials Insurance to cover the conduct of this trial.

**11. ADVERSE EVENTS**

**11.1 ADVERSE EVENT DEFINITION**

An adverse event is any untoward medical occurrence or an unanticipated benefit in a subject administered an investigational product, which does not necessarily have a causal relationship with this product. An adverse event can therefore be any unfavourable and/or unintended sign (including an abnormal laboratory finding), symptom or disease temporally associated with the use of an investigational product, whether or not related to the investigational product.

The causal relationship between an adverse event and the investigational product will be defined as below:

- **NOT RELATED** = an AE that does not follow a reasonable temporal sequence from the beginning of the subject’s investigational product and that can be reasonably explained by other factors, including underlying disease, complications, concomitant drugs, or concurrent treatment;
- **UNLIKELY RELATED** = an AE that follows a reasonable temporal sequence from the beginning of the subject’s investigational product administrationand that cannot be excluded as being possibly caused by the subject’s study participation;
- **POSSIBLY RELATED** = an AE that follows a reasonable temporal sequence from the beginning of the subject’s investigational product administrationand that can be excluded as being possibly caused by other factors, such as underlying disease, complications, concomitant drugs, or concurrent treatment;
- **RELATED** = an AE that follows a reasonable temporal sequence from the beginning of the subject’sinvestigational product administration, follows a known or hypothesized cause-effect relationship.

The severity of an adverse event will be recorded as one of the following:

- **MILD** = experience is minor and does not cause significant discomfort to subject or change in activities of daily living; subject is aware of symptoms, but symptoms are easily tolerated;
- **MODERATE** = experience is an inconvenience or concern to the subject and causes interference with activities of daily living, but the subject is able to continue with activities of daily living;
- **SEVERE** = experience significantly interferes with activities of daily living and the subject is incapacitated and/or unable to continue with activities of daily living.

Every reasonable effort will be made to follow up subjects who have adverse events.

**11.2 SERIOUS ADVERSE EVENT**

A serious AE (SAE) is any untoward medical occurrence experienced at any time during study participation that:

- results in death
- is life threatening
- requires in subject hospitalisation or prolongation of existing hospitalisation
- results in persistent or significant disability/incapacity (disability is defined as a substantial disruption of a person’s ability to conduct normal life functions)
- is a congenital anomaly/birth defect.

Important medical events that may not result in death, be life-threatening, or require hospitalisation may be considered a serious adverse product experience when, based upon appropriate medical judgement, they may jeopardise the subject and may require medical or surgical intervention to prevent one of the outcomes listed in this definition. Examples of such medical events include allergic bronchospasm requiring intensive treatment in an emergency room or at home, blood dyscrasias or convulsions that do not result in inpatient hospitalisation, or the development of drug dependency or drug abuse.

Instances of death or congenital abnormality, if brought to the attention of the Investigator at any time after cessation of the study and considered by the Investigator to be possibly related to use of the study products, will be reported to the Sponsor.

**11.3 DEFINITION OF LIFE THREATENING**

An adverse event is life threatening if the subject was at immediate risk of death from the event as it occurred, i.e. does not include a reaction that might have caused death if it had occurred in a more serious form. For instance, product induced hepatitis that resolved without evidence of hepatic failure would not be considered life threatening even though product induced hepatitis can be fatal.

**11.4 DEFINITION OF HOSPITALISATION**

Adverse events requiring hospitalisation should be considered serious. In general, hospitalisation signifies that the subject has been detained (usually involving an overnight stay) at the hospital or emergency ward for observation and/or treatment which would not have been appropriate at the study site. When in doubt as to whether hospitalisation occurred or was necessary, the adverse event should be considered as serious.

Hospitalisation for elective surgery or routine clinical procedures, which are not the result of an adverse event, need not be considered adverse events and should be recorded on a Clinical Assessment form and added to the CRF. If anything untoward is reported during the procedure, this must be reported as an adverse event and either ‘serious’ or ‘non-serious’ attributed according to the usual criteria.

**11.5 SERIOUS ADVERSE EVENT (SAE) REPORTING**

A copy of the SAE form provided in the investigator study master file should be completed as fully as possible. It is essential to enter the following information:

- Protocol and subject identifiers
- Subject’s demography
- Description of events, with diagnosis if available
- Investigator opinion of relationship to study product use
- Criterion for seriousness.

The following are desirable and are of particular relevance for investigator and BAT Medical Contact assessment of the SAE report:

- Date of onset of AE
- Date AE stopped, if relevant
- Study product use start date
- Study product use end date if relevant
- Action taken on study product use
- Outcome if known

The SAE form, completed as fully as possible must be e-mailed to the Medical Contact as soon as possible, but not later than 24 hours after study site personnel learn of the event. The Sponsors’ Primary Contact should also be notified of the situation by telephone or email.

The initial report will be followed up with more information as relevant, or as requested by the Medical Contact. This may require the Principal Investigator to obtain copies of hospital case reports, autopsy reports and other documents as applicable.

All SAEs will be reported to the REC as described in their Draft Guidance on the Use of Investigational Tobacco Products (September 2015).

**12. RISK / BENEFIT ASSESSMENT**

**Investigational site:**

- All staff involved in the study at the clinical site are fully experienced to perform each procedure involved in the study and will be trained appropriately before the start of the study if required. The site has previous experience in running pharmacokinetic and nicotine/tobacco product studies.

**Test Products:**

- Nicotine and tobacco use can have side effects, but subjects included in the study will already be using nicotine and tobacco products. During study product use, it is not expected that they would be exposed to nicotine levels higher than those they are usually exposed to during their daily consumption of nicotine and tobacco products. The duration of exposure for each product has been determined by the standard use of such products. Therefore, acute risks related to the product administration are anticipated to be low.
- Tobacco products and e-cigarettes may cause acute side effects, such as cough, irritation in the mouth and throat, palpitations, feeling faint, nausea, dizziness and headache. Although less common side effects are not anticipated in the study subjects, less common side effects include nasal congestion, stomach discomfort, hiccups and vomiting. Even less common are chest palpitations and cardiac arrhythmia and again these would be unanticipated in this study.

- Study participants will be notified in advance of giving their informed consent to participate in the study that:
- Nicotine and tobacco use can have side effects but as the subjects are already using tobacco products acute risks related to product administration are anticipated to be low. During study product use, the subjects are not likely to be exposed to nicotine levels higher than the ones subjects are usually exposed to during their daily consumption of tobacco products.
- E-cigarette solutions are contained within a cartridge which can only be used with the e cigarette provided. Minimal risk of skin exposure or accidental ingestion exists when using the e-cigarette, when exchanging cartridges or if cartridges are left unattended. Cartridges are childproof, and the nicotine solution cannot be extracted from the cartridge without using considerable force to break it open. The cartridge cannot be refilled. Subjects are not permitted to leave the study site with the investigational products at any time.
- Tobacco products, including cigarettes, are addictive. Consumption of tobacco products is associated with real risks of serious diseases.
- Electronic cigarettes may be hazardous to health and contain nicotine which is addictive.
- Electronic cigarettes are relatively new products. No information is available on the long-term effects of using electronic cigarettes, and this means there is uncertainty about the long-term effects of using these products on your health.
- The best way to avoid the risks associated with tobacco products and electronic cigarettes is not to use them at all.
- Each tobacco and nicotine product will meet the required quality standard applicable to marketed tobacco or nicotine products (as applicable).

**Procedures:**

- Taking blood and having a cannula inserted can be painful. Withdrawing blood is almost always a harmless procedure and for most people, taking a blood sample does not cause any serious problems. However, it may cause bleeding, bruising, discomfort, dizziness, infections and/or pain at the needle site. Some people may also feel faint or light-headed when blood is taken. Each procedure will be performed by specialised staff with experience and appropriate training in the respective techniques.

**Subject monitoring and follow-up:**

- The risk that subjects develop clinical symptoms is low as they will be administered products similar to those that they already use. All side effects will be monitored continuously, and medical treatment will be given if required. At each product use PK sessions and at the follow-up phone call, subjects will be actively prompted to report any Adverse Event (AE) they may have experienced since their discharge (prior to and during the product administration for the product use PK session) and all AEs will be recorded and followed-up appropriately. If the Principal Investigator withdraws a subject due to an AE (or the subject decides to withdraw), the subject will be followed-up until evaluations return to baseline.
- The recruitment of participants in the study is limited to those with effective contraception. Women will have a pregnancy test at the screening visit and admission, they will be withdrawn from the study if the test is positive.
- An Adverse Event Reporting Plan will be put in place before the start of the clinical phase to ensure the appropriate follow-up of the SAEs.
- If the Investigator (or the Sponsor) judges that it may be unwise to continue the study, the study will be terminated.

**Benefits:**

- There will be no medical advantages as a result of using the test products. However, the subjects will undergo a medical examination, which may provide them with information on their state of health.
- Subjects will be able to ask for advice to stop using tobacco / nicotine products and will be provided with a smoking cessation helpline number.

The results of the study will add to the current knowledge of nicotine delivery from e-cigarettes. This may help manufacturers to design better products which may help people to stop smoking conventional cigarettes.

**14. REFERENCES**

1. International Agency for Research on Cancer. (2007). IARC handbooks on cancer prevention, volume 11, Reversal of risk after quitting smoking. Lyon: International Agency for Research on Cancer.
2. Doll R, Peto R, Wheatley K, et al. (1994) Mortality in relation to smoking: 40 years' observations on male British doctors. BMJ, 309, 901-911.
3. Stratton K, Shetty P, Wallace R, & Bondurant S (eds.) (2001). Clearing the smoke: assessing the science base for tobacco harm reduction. Washington, DC: Institute of Medicine, National Academy Press.
4. World Health Organisation. (2011). WHO report on the global tobacco epidemic, 2011: Warning about the dangers of tobacco. Geneva: World Health Organization.
5. Mackay J & Eriksen M. (2002). The Tobacco Atlas, First Edition, pages 90-91. Geneva: World Health Organization.
6. Perfetti TA & Rodgman A. (2011). The Complexity of Tobacco and Tobacco Smoke. Beitrage Tabakforschung International, 24, 215–232.
7. Lunell E, Molander L, Ekberg K, Wahren. (2000). Site of nicotine absorption from a vapour inhaler – comparison with cigarette smoking. Eur J Clinical Pharmacol, 55,737-741.
8. Goniewicz ML, Knysak J, Gawron M, et al. (2014). Levels of selected carcinogens and toxicants in vapour from electronic cigarettes. Tob Control, 23, 133-139. doi: 10.1136/tobaccocontrol-2012-050859.
9. McRobbie H, Phillips A, Goniewicz ML, et al. (2015). Effects of switching to electronic cigarettes with and without concurrent smoking on exposure to nicotine, carbon monoxide, and acrolein. Cancer Prev Res, 8, 873-878. doi: 10.1158/1940-6207.
10. Nutt DJ, Phillips LD, Balfour D, et al. (2014). Estimating the harms of nicotine-containing products using the MCDA approach. Eur Addict Res, 20, 218-225. doi: 10.1159/000360220.
11. McNeil A, Brose LS, Calder, et al. (2015). E-cigarettes: an evidence update A report commissioned by Public Health England. Retrieved from https://www.gov.uk/government/uploads/system/uploads/attachment_data/file/457102/Ecigarettes_an_evidence_update_A_report_commissioned_by_Public_Health_England_FINAL.pdf.
12. Farsalinos KE & Le Houezec J. (2015). Regulation in the face of uncertainty: the evidence on electronic nicotine delivery systems (e-cigarettes). Risk Manag Healthc Policy, 8, 157-167. doi: 10.2147/RMHP.S62116.
13. Royal College of Physicians. Nicotine without smoke: Tobacco harm reduction. London: RCP, 2016.
14. Brown J, Beard E, Kotz D, et al. (2014). Real-world effectiveness of e-cigarettes when used to aid smoking cessation: a cross-sectional population study. Addiction, 109, 1531-1540. doi: 10.1111/add.12623.
15. Brose LS, Hitchman SC, Brown J, et al. (2015). Is the use of electronic cigarettes while smoking associated with smoking cessation attempts, cessation and reduced cigarette consumption? A survey with a 1-year follow-up. Addiction, 110, 1160-1168. doi: 10.1111/add.12917.

**15. APPENDICES**

**APPENDIX 1****: Fagerstrom Test for NICOTINE Dependence**

This questionnaire will be administered during screening.

For each statement, circle the most appropriate number that best describes you.

| 1. How many cigarettes do you smoke per day? |
| --- |
| a) 10 or less |
| b) 11 – 20 |
| c) 21 – 30 |
| d) 31 or more |
|  |
| 2. How soon after you wake up do you smoke your first cigarette? |
| a) 0 – 5 min |
| b) 6 – 30 min |
| c) 31 – 60 min |
| d) After 60 min |
|  |
| 3. Do you find it difficult to refrain from smoking in places where smoking is not allowed (e.g. hospitals, government offices, cinemas, libraries etc)? |
| a) Yes |
| b) No |
|  |
| 4. Do you smoke more during the first hours after waking than during the rest of the day? |
| a) Yes |
| b) No |
|  |
| 5. Which cigarette would you be the most unwilling to give up? |
| a) First in the morning |
| b) Any of the others |
|  |
| 6. Do you smoke even when you are very ill? |
| a) Yes |
| b) No |
|  |

**APPENDIX 2: Tobacco use history questionnaire**

This questionnaire will be administered during screening.

What types of tobacco products do you smoke or use, even if only occasionally? Please select all that apply.

|  Manufactured cigarettes  Roll your own  Pipe tobacco  Shisha/Hookah  Cigarillo/Little cigars  Cigars  Other, namely... |
| --- |

For how long have you been smoking? Please select one answer.

|  Less than 1 month  Between 1-6 months  Between 7-12 months  Between 1-2 years  Between 2-5 years  Between 5-10 years  Between 10-20 years  More than 20 years |
| --- |

How many standard cigarettes do you currently smoke? Please enter a value between 1 and 60 below for the number of cigarettes and a time frame (per day / per week / per month)

|  |
| --- |

What type of cigarettes do you currently smoke most? Please describe these cigarettes including the brand, the colour of the packet, and any other descriptors such as slim, menthol, superking etc.

**APPENDIX 3: E-cigarette use history questionnaire**

This questionnaire will be administered during screening.

What types of e-cigarettes do you use, even if only occasionally? Please select all that apply.

| none  cigarette-like  e-cigarette loaded with pre-filled cartridges of e-juice  e-cigarette with a tank which you fill manually from a separate bottle of e-juice  Other, namely... |
| --- |

For how long have you been using e-cigarettes? Please select one answer.

|  Less than 1 month  Between 1-6 months  Between 7-12 months  Between 1-2 years  Between 2-5 years  5+ years |
| --- |

How often do you use e-cigarettes?

never

daily

once or twice a week

less than once a month

once or twice a month

**APPENDIX 4: Product satisfaction questionnaire**

Subjects will answer these questions during PK sessions by drawing a vertical line on 100mm horizontal line provided below.

**Can you tell me how much you like this nicotine product?**

**Very much Not at all**

**APPENDIX 5: Clinical Laboratory Panel for Screening**

| **Serum biochemistry:** | **Haematology:** |
| --- | --- |
| Aspartate aminotransferase (AST) | Haemoglobin |
| Alanine aminotransferase (ALT) | Haematocrit (packed cell volume [PCV]) |
| Alkaline phosphatase | Total and Differential leukocyte count |
| Gamma‑glutamyl transferase (GGT) | Red blood cell (RBC) count |
| Sodium | Platelet count |
| Potassium | Mean cell volume (MCV) |
| Chloride | Mean cell haemoglobin (MCH) |
| Calcium | MCH concentration (MCHC) |
| Inorganic phosphate | **Urinalysis:** |
| Glucose (fasting) | Microscopic examination^e^ |
| Urea | pH |
| Uric acid | Specific gravity |
| Total bilirubin | Protein |
| Direct bilirubin | Glucose |
| Creatinine | Ketones |
| Total protein | Bilirubin |
| Albumin | Blood |
| Total cholesterol | Nitrite |
| Triglycerides | Urobilinogen |
| Creatinine phosphokinase (CPK) | Leukocytes |
| Blood urea nitrogen (BUN) | Urine pregnancy test**^c^** |
| **Serology:** | Urine drug and alcohol screen^d^ |
| Hepatitis B surface antigen (HBsAg) |  |
| Hepatitis C antibody |  |
| Human immunodeficiency virus (HIV)**^b^** |  |
| **Hormone Panel:** |  |
| Follicle‑stimulating hormone (FSH) **^a^** |  |
| Human chorionic gonadotropin (hCG; serum pregnancy test)^f^ |  |

^a^ To confirm post-menopausal status

**^b^**HIV1/2 and p24 antigen

**^c^** In all females. A positive urine pregnancy test will be confirmed with a serum pregnancy test.
^d^ Urine drugs of abuse and alcohol screen will be conducted for Amphetamine, Barbiturate, Benzodiazepine, Cannabinoid (for tetrahydrocannabinol), Cocaine, Methadone and Opiate (for Morphine).

**^e^** To be performed upon significant findings on the macroscopic examination

^f^ In females of child bearing potential only

1. At completion of final Study PK session (Day 8) [↑](#footnote-ref-1)
2. Symptom-driven physical examination will be performed if deemed necessary. [↑](#footnote-ref-2)
3. Lung function test to be carried out during the screening period (Day-28 to Day -1). [↑](#footnote-ref-3)
4. Urine Alcohol. [↑](#footnote-ref-4)
5. Female subjects only. In serum at screening and in urine on admission and discharge.

   ^5^ Use count, puff count, cartridge weight for e-cigarette (Arms C-H) use only; MLE for Arm (A & B) only

   ^6^ -5 minutes, 15 minutes and 20 minutes [↑](#footnote-ref-5)
6. For a breakdown of the clinical laboratory panel for screening, see Appendix 5. [↑](#footnote-ref-6)
7. Urine cotinine level ≥200 ng/mL. [↑](#footnote-ref-7)
8. International Organization for Standardisation. [↑](#footnote-ref-8)
9. See Appendix 2 for tobacco and e-cigarette use history questionnaires. [↑](#footnote-ref-9)
10. For a breakdown of the clinical laboratory panel for screening, see Appendix 5. [↑](#footnote-ref-10)
11. For a breakdown of the clinical laboratory panel for screening, see Appendix 3. [↑](#footnote-ref-11)
